# PamR, a new MarR-like regulator affecting prophages and metabolic genes expression in *Bacillus subtilis*

**DOI:** 10.1371/journal.pone.0189694

**Published:** 2017-12-14

**Authors:** Alba De San Eustaquio-Campillo, Charlène Cornilleau, Cyprien Guérin, Rut Carballido-López, Arnaud Chastanet

**Affiliations:** 1 MICALIS, INRA, AgroParisTech, Université Paris-Saclay, Jouy-en-Josas, France; 2 MaIAGE, INRA, Université Paris-Saclay, Jouy-en-Josas, France; Centre National de la Recherche Scientifique, Aix-Marseille Université, FRANCE

## Abstract

*B*. *subtilis* adapts to changing environments by reprogramming its genetic expression through a variety of transcriptional regulators from the global transition state regulators that allow a complete resetting of the cell genetic expression, to stress specific regulators controlling only a limited number of key genes required for optimal adaptation. Among them, MarR-type transcriptional regulators are known to respond to a variety of stresses including antibiotics or oxidative stress, and to control catabolic or virulence gene expression. Here we report the characterization of the *ydcFGH* operon of *B*. *subtilis*, containing a putative MarR-type transcriptional regulator. Using a combination of molecular genetics and high-throughput approaches, we show that this regulator, renamed PamR, controls directly its own expression and influence the expression of large sets of prophage-related and metabolic genes. The extent of the regulon impacted by PamR suggests that this regulator reprograms the metabolic landscape of *B*. *subtilis* in response to a yet unknown signal.

## Introduction

*B*. *subtilis*, the model for gram-positive bacteria, has been studied for decades for its fundamental cellular processes and regulatory pathways such as transcription, chromosome segregation, metabolism, cell growth and division, but also for its distinctive cellular differentiation (or developmental) programs: competence for DNA uptake (K-state), sporulation and biofilm formation. While the fundamental processes were studied during steady state, i.e. in an expected unchanging environment and unvarying physiological state, the differentiation programs take place during the stationary phase of growth. In fact, these “late” programs, especially competence and sporulation, are induced by the transition from abundance to exhaustion of nutriments and reveal how bacteria adapt to changing environments by completely reprogramming their gene expression (for review on these programs see [1–5]). It is important to stress that adaptation is a carryall concept because it involves, by definition, everything that is not steady state, hence entry into and exit from stationary phase, modification of the carbon sources and growth regime, but also any sort of stress coming from physical (temperature, pressure, light…) or chemical (ions, drugs…) modifications.

Among the many responses evolved by bacteria to cope with environmental changes, the MarR (Multiple antibiotic resistance Regulator) family of transcription regulators (TRs) is widespread both in bacteria and archea. Their abundance is correlated with the diversity of lifestyles encountered by each species [6]. They are involved in a wide variety of adaptations such as to oxidative stress or resistance to antibiotics (via expression of multidrug efflux pumps), but also catabolic control or expression of virulence factors [7]. Expression of the genes encoding MarR-like regulators is submitted to a diversity of regulatory mechanisms but are often autoregulated [6–8]. In contrast to such diversity, they share a strong structural homology, with a characteristic winged helix-turn-helix (wHTH) DNA-binding domain. Their activation is usually mediated through the binding of a ligand, inducing a conformational change that alters their structural properties, and consequently their DNA-binding abilities [7, 9].

In addition to the dramatic changes that lead to specific stress responses, adaptation to environmental challenges involves several, positive and negative, transcriptional regulators (TR). They are usually referred to as either transition state regulators, kicking off or inhibited at the exit point from exponential phase of growth (AbrB, Hpr/ScoC, Abh…) [10–13], or stationary phase regulators, including the master regulators controlling the main developmental processes of these stages, such as competence and sporulation (ComK, Spo0A, SigH, slrR) [14–16]. Note that this discrimination does not reflect bona fide well-defined categories, their regulation pathways being intimately intricated.

Among them, AbrB has probably been the most studied transition state regulator since it was discovered as a suppressor of defects in sporulation deficient mutants [16]. The protein controls the expression of over a hundred genes, either directly mainly by repressing them during active growth, or indirectly by affecting the expression of other regulators (ScoC, SigH, Abh and SinR) [16, 17]. Target genes present a wide panel of functions including sporulation, competence, extracellular degradative enzymes, nitrogen phosphate and amino acid metabolism, motility, synthesis of antibiotics, resistance to oxidative stress. Albeit not essential, AbrB allows a global reprogramming of the genetic expression that gives the cell an increased fitness in a changing and depleted environment.

Not surprisingly, the transition from fast to slow growing conditions also involves sensors of the nutritional state of the cell, such as CodY, that control carbon metabolism [11, 18]. During growth, CcpA is one of the main regulators of carbon metabolism, acting positively and negatively on the expression of many genes, especially those allowing acquisition of the much-preferred *B*. *subtilis* carbon source, glucose [11, 19]. Many other regulators (e.g. TnrA, CcpC, GltC), metabolites and secondary messengers are involved as well in the fine tuning of the metabolic state of the cells and their adaptation to changes in availability of carbon sources [11].

Finally, another known consequence of stress is the induction of prophages and mobile genetic elements as ICE*Bs1*. DNA damage has been the most studied and is the best characterized mechanism that induce their activation [20, 21] but a number of other stresses (including membrane perturbation or oxidative stress) have been shown to activate them as well [22–24]. Thus, adaptation to the environment is a complex phenomenon involving a large range of mechanisms through many different regulators, which can optimize the response and consequently the fitness of the bacteria to changing conditions.

Here, we report the characterization of an unknown operon in *B*. *subtilis* encoding YdcH a putative transcriptional regulator of the MarR family. We showed that the *ydcFGH* operon is under the control of two promoters, and that one of them is directly repressed by YdcH. Furthermore, we provide evidence that YdcH positively and negatively affects the expression of a large set of genes, mainly encoding metabolic and prophage-related proteins, and numerous transcriptional regulators. Together, our results suggest that YdcH, which we propose to rename PamR (for Prophages and Metabolic Regulator), is a transcriptional regulator in *B*. *subtilis* that may be required for adaptation to a yet to be discovered condition.

## Materials and methods

### Bacterial strains and growth conditions

*Escherichia coli* DH5α strain was grown in LB medium and transformed with standard procedures with ampicillin (100 mg.ml^-1^) or kanamycin selection (25 mg.ml^-1^). All *Bacillus subtilis* but strain 3725 [25] are derivatives of the laboratory collection strain 168 (designated 168-Oxford on S1 Table) and were grown at 37°C in the rich media Lysogeny Broth (LB), or Sterlini and Mendelstam (CH) [26]. To assay growth proficiency, wild type and mutant strains were additionally grown on poor defined CE [25] and S [27] media. Mutants deficient for *mreB* were grown with media supplemented with 20 mM MgSO_4_. *B*. *subtilis* transformations were performed using the one-step method mainly as described previously [28]. All plasmids used except pDR244 were replicative in *E*. *coli* but not in *B*. *subtilis*, and transformants in the latter involved integration of the constructs into the chromosome. Double cross-over integrations at the *amyE* or *sacA* loci of *B*. *subtilis* were confirmed by PCR amplification using oligonucleotides flanking the cloned areas, and were systematically sequenced (Eurofins MWG). Strains obtained from the *B*. *subtilis* Genetic Stock Center (BGSC) were backcrossed into our 168 background (see S1 Table). Antibiotics for clone selections were used at the following concentrations: chloramphenicol (cm), 5 μg/ml; kanamycin (kan), 20 μg/ml; spectinomycin (spc), 100 μg/ml; erythromycin (erm), 1 μg/ml. Growth curves and luciferase assays were performed in 96-well plates on a Synergy2 microplate reader (Biotek) with maximum agitation at 37°C and optical density (OD600 nm) and relative luminescence units (RLU) were measured in real time during growth. The luciferase activity is reported as normalized luminescence corresponding to the ratio of the normalized RLU by the normalized OD600nm.

### Disc diffusion assay

Disk diffusion assays were performed as follows. Square Petri dishes containing 70 mL of LB agar medium were covered with 10 mL of soft agar (0.7% agar in LB) in which 200 μL of exponentially growing cells of the strain being tested were resuspended. The plates were allowed to dry for 30 min in a laminar flow hood before 6 mm paper disks containing 10 μl of antibiotic solution at the following concentrations were placed on the plates: vancomycin 10mg/mL, bacitracin 200 mg/mL, methicillin 1 mg/mL, polymixine 20 mg/mL, erythromycin 10 μg/mL, tetracycline 1 mg/mL, ciprofloxacine 10 μg/mL, levofloxacine 10 mg/mL, rifampicin 10 mg/mL. The plates were incubated at 37°C overnight before measuring the zones of inhibition.

### Plasmids construction

Strains, plasmids and oligonucleotides used are listed in S1, S2 and S3 Tables, respectively. We constructed suicide plasmids for chromosomal integration at the *amyE* locus of *B*. *subtilis* of transcriptional fusions between *lacZ* and different fragments of the *ydcF* promoter, as follow: DNA fragments containing the putative P_2_ promoter, both P_1_ plus P_2_, or none (“P_0_”) were PCR-amplified using purified DNA from WT strain 168 as template and pairs of oligonucleotides AC1242/AC1243, AC1240/AC1242 and AC1243/AC1241, respectively; these fragments were subjected to *EcoR*I/*BamH*I digestions and cloned into the corresponding sites of digested plasmid pDG1728 (containing the reporter gene *lacZ*), generating pAC772, pAC775 and pAC778, respectively. The DNA fragment containing only the P_1_ putative promoter was cloned similarly using oligonucleotides AC1240/AC1241 but the resulting fragment was sub-cloned into the *EcoR*I/*BamH*I digested pDG1663, to give plasmid pAC769. A plasmid allowing integration at the *sacA* locus of this last transcriptional fusion was generated by PCR amplifying a DNA fragment using oligonucleotides AC1240/AC1280. The *EcoR*I/*HinD*III digested fragment was cloned in the corresponding sites of pSac-Cm, giving pAC826.

A transcriptional fusion between the reporter operon *luxABCDE* from *Photorhabdus luminescence*, optimized for *B*. *subtilis* [29], and the promoter P_*ydc1*_ of *ydcF* was generated by cloning a *Spe*I/*EcoR*I digested PCR fragments amplified with oligonucleotides AC1240/AC1287, into plasmid pAH328. This fragment (P_*ydc1s*_, “s” standing for short) was devoid of any part of the *ydcF* orf, containing only the putative transcriptional informations. The resulting suicide vector pAC834 allowed integration of the reporter fusion as a single copy at the *sacA* locus of *B*. *subtilis*.

To purify PamR (YdcH) from a heterologous expression *E*. *coli* host (BL21), a pET28a-derivative expression plasmid was generated. For this, a DNA fragment was obtained by PCR using oligonucleotides asec4/asec64, restriction digested using *Nco*I/*Bam*H1, and sub-cloned (into DH5a) into the corresponding sites of expression vector pET28a (Novagen). The resulting pET_ydcH_6H-Nt was sequenced and transformed into the recipient expression host, freshly prior to expression and purification.

### Strain constructions

A mutant for *ydcH*, ABS1381, was constructed in which most of the orf (124 of the 147 codons, starting at codon 15) has been replaced by a spectinomycin resistance cassette (*spc*). For this, a DNA fragment was generated by OE-PCR (using primers AC1052/AC1053 and AC1054/AC1055) containing *spc* flanked by the up and downstream regions (450 to 490 bp long) of the *ydcH* locus, and transformed into the wild type 168 *B*. *subtilis* strain. Upon transformation into *B*. *subtilis* the resistant clones were checked for the replacement of the gene and sequenced using primers AC1056 and AC1057 to insure any introduction of sequence mutations into the amplified flanking areas.

Markerless deletions were generated by transforming BKE derivative strains, in which the deleted genes are replaced by an erythromycin cassette flanked by *loxP* site, by pDR244. pDR244 is a thermosensitive vector encoding the Cre recombinase and allowing excision of DNA fragments framed with *loxP* sites [30].

### Protein purification

PamR was expressed from a BL2I derivative *E*. *coli* strain grown to mid exponential growth, in the presence of selective pressure, prior to induction with IPTG (1 mM final) for 3h at 30°C. Harvested cells were resuspended in 35 mL of W buffer (Tris-HCl 20 mM pH7, KCl 500 mM, imidazole 25 mM, glycerol 10%) supplemented with lysozyme (0.25 mg.ml^-1^) and cOmplete^tm^ protease inhibitor cocktail (Roche), and disrupted by sonication. Cell debris were removed by centrifugation for 20 min at 17000 g and the resulting crude extract was loaded on a 1 mL Ni-NTA agarose (Qiagen) column equilibrated with buffer W. The column was washed with 30 volumes of buffer W, and the protein eluted with sequential addition of 2 mL of buffer E1 (identical to W but imidazole was 100 mM) then E2 (identical to W but imidazole was 300 mM). Fractions containing >95% pure PamR were pooled and dialyzed twice against dialysis buffer (NaPO4 50 mM, NaCl 300 mM, glycerol 50%) before freezing at -20°C until further use.

### Electrophoretic mobility shift assay (EMSA)

A fluorescently labeled DNA probe corresponding to the promoter of *ydcF* was generated by PCR using Phusion polymerase (NEBiolabs) and Cy5-labeled oligonucleotides ac-1391/ac-1392. The DNA probe containing a mutated IR1 sequence (IR1*) was generated similarly but in two sequential steps. First, we generated two PCR fragments overlapping at the IR site using oligonucleotides ac-1390/ac-1386 and ac-1387/ac-1385; ac-1386 and ac-1387 containing the desired mutations. Next, an OE-PCR was performed using the two resulting DNA fragments as templates and Cy5-labelled oligonucleotides ac-1391/ac-1392, resulting in a fluorescent probe containing the mutated inverted repeat site.

Binding was performed in the presence of binding buffer (20 mM NaPO4 pH7, 50 mM NaCl, 5 mM MgCl2, 10% glycerol) by incubating 0.15 to 2.5 pmol of purified PamR protein to 0.1 pmol of labeled DNA probe (wt or mutant for IR1) in the presence of an excess of non-specific DNA (0.1 μg/μl salmon sperm DNA). After 10 min of binding at room temperature, samples were loaded on a 4% native acrylamide gel and run at room temperature for 80 min. Fluorescence was detected directly on gel using a ChemiDoc XRS+ system (Biorad).

### Complete genome re-sequencing

200 ng of chromosomal DNA of exponentially grown *B*. *subtilis* was sent to GATC Biotech SARL (Mulhouse) for generation of a genomic library and next-generation sequencing, using Illumina technology, with paired-ends, 125 bp long reads and 5 million of read pairs. A pre-analysis (with semi-automatic detection and mapping) with mapped SNPs and InDels was delivered. We further analyzed the data with the Tablet software [31].

### Genome-wide transcriptome profiling by RNAseq

200 mL cultures of the control (ABS2005) and Δ*ydcH* (ASEC56) strains were inoculated at an OD_600nm_ 0.005 from overnight cultures in CH medium, and grown at 37°C with maximum aeration. At OD_600 nm_ = 0.2, 70 mL samples were collected at mid-exponential (OD_600nm_ 1.5) growth and instantly mixed with 30 mL ice-cold killing buffer (20 mM Tris-HCl pH = 7.5; 5 mM MgCl_2_, 20 mM NaN_3_), centrifuged 10 min at 4700 rpm and 4°C and pellets were frozen in liquid nitrogen and save at -80°C until further used. Frozen materials were resuspended in 200 μL ice-cold killing buffer (20 mM Tris-HCl pH = 7.5, 5 mM MgCl_2_, 20 mM NaN_3_), 500 μL of small glass beads and 1 mL of lysis buffer (4 M guanidine-thiocyanate, 25 mM sodium acetate pH = 5.2, 0.5% N-lauroylsarcosinate). Cells were disrupted with a Fastprep (MP Biomedicals) at 4°C for 30 sec at maximum power and centrifuged at maximum speed for 3 min at 4°C. RNA were extracted by standard Phenol/Chloroform extraction and precipitated in the presence of isopropanol. RNA pellets were resuspended in 75 μL H_2_O for 3 h at 4°C, treated with QIAGen RNase-Free DNase and cleansed with Norgen Concentration Micro Kit as per manufacturer’s instructions. RNA libraries and next generation sequencing were performed on RNA samples obtained from 3 completely independent experiments, and benefits from the expertise of the High-throughput Sequencing Platform of I2BC (CNRS, Gif/Yvette, France). Sequencing was performed using single read 75 bases with 30 million stranded reads per sample on a NextSeq 500 Illumina sequencer (GEO accession number GSE104816).

### RNAseq data analysis

Three prime end low quality nucleotides were firstly removed from reads using Trimmomatic (version 0.36; with options -TRAILING:20) [32] then trimmed using Sickle (https://github.com/najoshi/sickle) (version 1.33; with options -n -x -q 30 -l 30). Read mapping against the reference genome of *B*. *subtilis* strain 168 (AL009126; https://www.ncbi.nlm.nih.gov/nuccore/AL009126.3) was performed using Bowtie 2 (version 2.2.6; with options -N 1 -L 16 -R 4) [33]. Read count was performed on annotated genes of the reference genome using HTSeq count (version 0.6.0; with default options) [34]. Gene aggregated read counts are available on GEO (accession # GSE104816). Differential expression analyses were performed using EdgeR package based on an over-dispersed Poisson model applied to the 3 biological replicated count data and empirical Bayes methods used to moderate the degree of overdispersion across transcripts [35]. Control of the False Discovery Rate relied on q-values obtained with R package FDRtools [36]. We considered that up-regulated native expression segments were affected by the YdcH mutantion if the signal exhibited differential expression according to the specified amplitude (effect ≥ log2(2)) and false discovery rate (q-value ≤ 0.05) cut-offs. Reciprocally, down-regulation was considered detected when effect ≤ -log2(2), q-value ≤ 0.05.

## Results

### A strain mutant for *mreB* harboring multiple mutations

**Fig 1:**
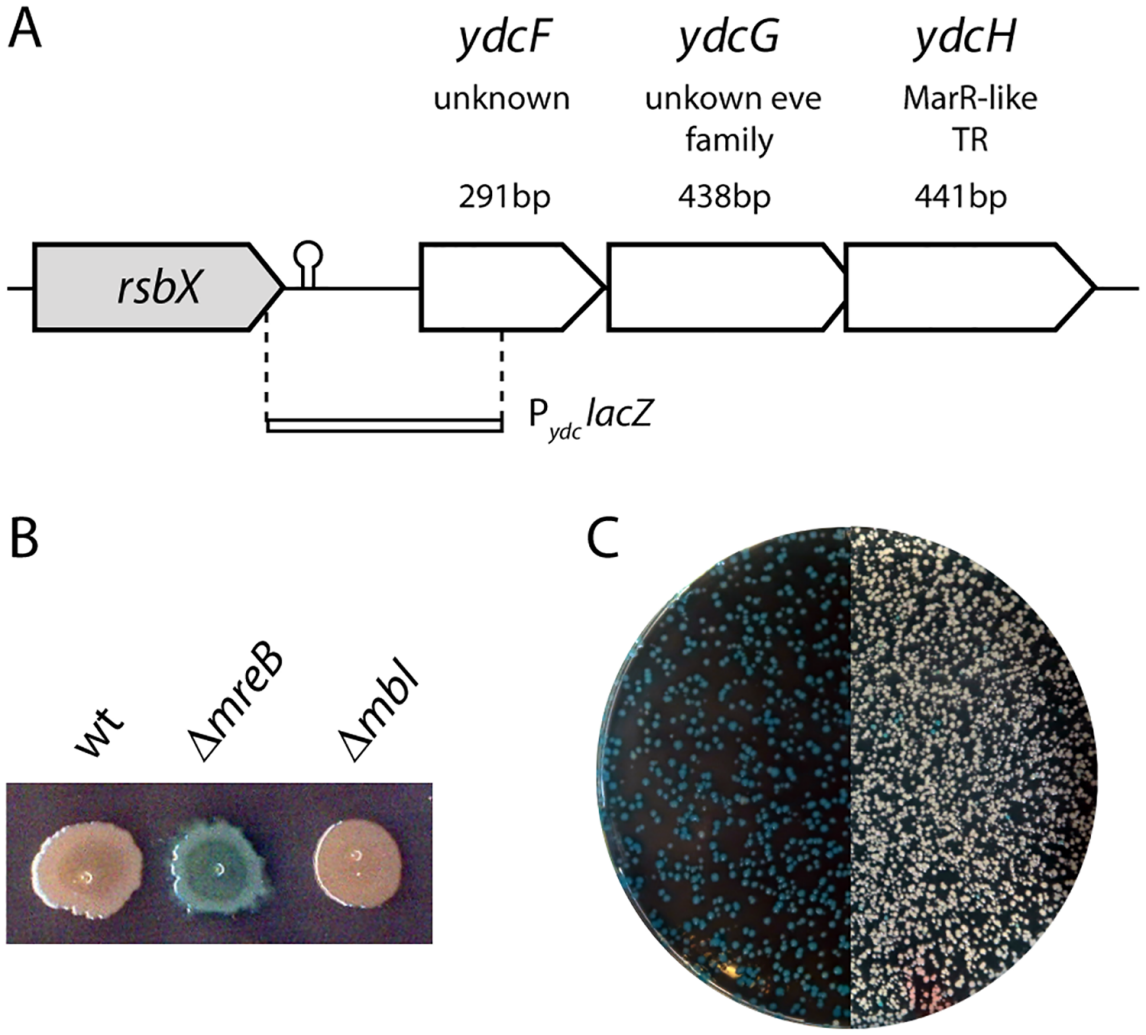
*ydcFGH*, an operon of unknown function induced in a Δ*mreB* strain. A. Schematic representation of the genetic organization of the *B*. *subtilis ydcFGH* locus. Gene size of the orfs and putative functions are indicated above each gene. “P_*ydc*_ *lacZ*” shows the approximate size and localization on the locus of the DNA fragment amplified to construct the transcriptional reporter fusion to *lacZ* (strain ABS1761). B. A P_*ydc*_ *lacZ* fusion is induced in a strain lacking *mreB* (3725 “Δ*mreB”*; ABS1762) but not *mbl* (Δ*mbl*; ABS1769) nor in its wild type parent (Wt; ABS1761). C. Transformation of chromosomal DNA from strain ABS1761 (*amyE*::P_*ydc*_ *lacZ-spc*) into the recipient 3725 (neo- Δ*mreB*) leads to 100% of the spectinomycin/kanamycin resistant colonies expressing the *lacZ* reporter fusion (left) while the reverse transformation (right) leads to a limited number of blue colonies, indicating the absence of genetic link between Δ*mreB* and the factor inducing the reporter.

In the course of a whole genome transcriptional analysis (unpublished results), we noticed the high induction of three transcripts, *ydcF*, *ydcG* and *ydcH*, in a published strain of *B*. *subtilis* deleted for *mreB* (3725) [37] but, intriguingly, not in a mutant inactivated for its paralog *mbl*. Interested in uncovering the reasons of such dissimilarity between the mutants, we decided to study the regulation of these genes and to uncover their putative function. To confirm our original observation that all three genes are strongly induced in a strain inactivated for *mreB* (3725), we created a transcriptional fusion between *lacZ* and the region upstream of *ydcF* (P_*ydc*_ *lacZ*), that extended from the upstream *rsbX* gene up to the middle of *ydcF* (Fig 1A), and introduced it at the ectopic *amyE* locus. As expected, the fusion was not induced when placed in a wild-type background (ABS1761) nor when combined with a deletion of *mbl* (ABS1769), but was strongly induced when introduced into the Δ*mreB* strain 3725 (ABS1762)(Fig 1B), confirming our original observation. However, when, in a reversed strategy, the chromosomal DNA of strain 3725 was transformed into ABS1761 containing the reporter and selected for the neomycin resistance marker associated to the *mreB* deletion, the fusion was intriguingly not induced in the vast majority of the neomycin resistant transformants (Fig 1C, right). We deduced from this that the locus responsible for the induction of the reporter in ABS1762 was not genetically linked to *mreB*, suggesting the presence of an extragenic mutation in strain 3725.

Using next generation sequencing, we completely sequenced the genome of this mutant, along with that of the wild type 168 strain. We found 24 variations common to the 3725 mutant and its parental wild type strain 168 relative to the published sequence of *B*. *subtilis subsp*. *subtilis str*. *168* (GenBank AL009126.3)(Table 1 and S4 Table), indicating a polymorphism between wild types. Most mutations appeared to be silent or affecting untranslated regions. However and unexpectedly, the sequencing revealed the overwhelming presence of 51 sequence variations in the mutant relative to its parental 168 wild type strain. Half of these mutations were previously reported in a mutant of *B*. *subtilis* forming L-forms (PDC134) [38]. Among the uncovered mutations, we noticed a frameshift in *ydcH* leading to a premature stop codon, cutting off more than half of the resulting protein. Since YdcH shares significant similarities with the MarR family of transcriptional regulators, we hypothesized that this protein could regulate the expression of the *ydcF*, *ydcG* and *ydcH* genes.

**Table 1:** Sequence variations detected in strain 3725.

Variations common to the parental (wt) and 3725 strains (relative to the published 168 sequence)	Variations in Δ*mreB* 3725 relative to its parental wild type 168 strain	
	Variations in strain 3725 also reported in strain PDC134	Variations unique to strain 3725
INTRAGENIC	INTRAGENIC	INTRAGENIC
*uvrX*	*sepF*	*parC*
*rrnI-16S*	*walR*	*rRNA23S*
*trnI-asn*	*ytpS* (= *sftA*)	*ylyB*
*rrnG-16S*	*epsC*	*trmD*
*rrnB-16S*	*comP*	*pksN*
*pstS* (5’UTR)	*sigI*	*oppD*
*ydzN* (5’UTR)	*bscR* (= *FatR*)	*gerAA*
*yjpA* (3’UTR)	*ymfD*	*s1255*/*sspG*
*yjpA* (3’UTR)	*mmgA*	*accC*
*zwf* (ter)	*yuxG* (= *rahEW*)	*glcK*
*zwf* (ter)	*ydcH*	*gltA*
*zwf* (ter)	*yhgE*	*sacA*
*zwf* (ter)	*yisQ* & *S389*	*ilvC* & *s1070*
*ywbD* (ter)	*yjcM* & *S430*	*yoqA*
synonymous codon (silent)	*BSU_misc_RNA_20*	*yqbS*
*uvrX*	*panE*	*yesY*
*uvrX*	*BSU_misc_RNA_28*	*yutE*
	*yxbD*	synonymous codon (silent)
		*pgdS*
		*rluB*
		*yqeZ*
		*ygaN*
		*yozT*
INTERGENIC	INTERGENIC	INTERGENIC
int (up *trnI-asn*)	*s1123* (5’utr *NifZ)*/*s1124* (5’utr *braB*)	int *bsrB*/*yrvM*
int (up *trnI-asn*)	*s1145* (5’ utr y*tnP*)	*s1101* (5’ utr *CitZ*)
int (dn *trnI-gly*)	*s125* (5’ utr *hxlR*)	*s1101* (5’ utr *CitZ*)
int (dn *trnI-ala*)	*s486* (5’ UTR *mtnE*)	
int (*s1555*/*purA*)	int *yorN*/*yorM*	
int (*yocK*/*azoR1*)	*s352* (3’ Utr *yhaH*)	
*s1417*	*s736* (dnstream *yocJ*)	
int (*ydgF*/*dinB*)	*s596* (5’utr *ylxY*)	

### Expression of *ydcFGH* is driven by two promoters

**Fig 2:**
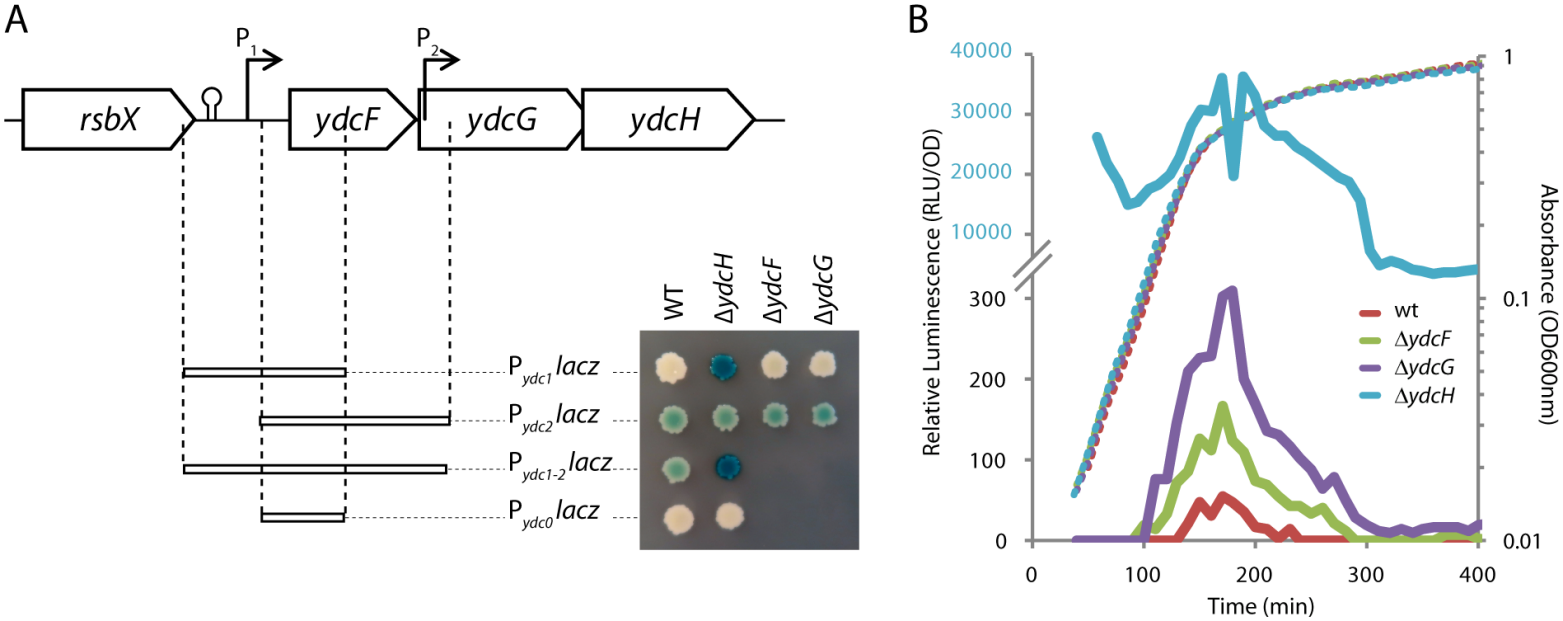
The expression of *ydcFGH* is driven by two promoters. A. Schematic representation of the DNA fragments of the *ydcFGH* locus used for generating *lacZ* reporter fusions. The two putative promoters are indicated by arrows and the names of the resulting transcriptional fusion to *lacZ* are indicated below. On the right is displayed a picture of an X-Gal-LB plate to visualize LacZ activity of colonies harboring *lacZ* transcriptional fusions to P_*ydc1*_, P_*ydc2*_, P_*ydc1-2*_ or P_*ydc0*_ placed in either WT (ABS1761; ABS1763; ABS1765; ABS1767, respectively), or Δ*ydcH* (ABS1820; ABS1821; ABS1822; ABS1823, respectively), and to P_*ydc1*_ or P_*ydc2*_ in Δ*ydcF* (ASEC297; ASEC333) or Δ*ydcG* (ASEC301; ASEC335) background. B. Expression of a P_*ydc1*_ *luxABCDE* transcriptional fusion in cells grown in LB medium, in a wild type (red; ABS2005) or mutant for *ydcF* (green; ASEC325), *ydcG* (purple; ASEC327) or *ydcH* (blue; ASEC329) background. Note that the Δ*ydcH* data are relative to the upper part of the ordinate axis (in blue). Growth curves are presented as dotted lines and correspond to the optical density at 600nm while luciferase activities (plain lines) are relative luminescence units normalized by the OD_600nm_.

The systematic mapping of transcription units of *B*. *subtilis* in a broad variety of conditions [39] has shown that the *ydcF*, *ydcG* and *ydcH* genes are expressed as two transcripts, suggesting they are organized as an operon expressed from two promoters. One, presumably located in front of *ydcF* (hereafter named P_*ydc1*_), that would initiate the expression of a long transcript including the three open reading frames (orf), and a second (P_*ydc2*_) lying at the beginning of the *ydcG* coding region and allowing expression of *ydcG* and *ydcH* (Fig 2A). In order to characterize these two putative promoters, we constructed three transcriptional fusions between *lacZ* and the region containing only the second putative promoter (P_*ydc2*_*lacZ*; ABS1763), both promoters (P_*ydc1-2*_*lacZ*; ABS1765) or the region in between them (P_*ydc0*_*lacZ*; ABS1767) (Fig 2A), in addition to ABS1761 carrying only the first promoter (P_*ydc1*_*lacZ*).

In WT cells growing in rich LB medium, no expression could be detected from P_*ydc0*_ and P_*ydc1*_, while a weak expression could be observed with P_*ydc2*_ and P_*ydc1-2*_, suggesting that only the second promoter is active in rich medium (Fig 2A). This is in agreement with the transcriptional study from Nicolas and co-workers that showed that the level of *ydcF* transcripts are close to the threshold of detection in most growth conditions, while *ydcG* and *ydcH* transcripts, which strongly correlate with each other, are expressed at basal albeit significant levels [39].

Because we observed a frameshift mutation in *ydcH* in strain 3725 in which P_*ydc1*_ was strongly activated, we wondered if YdcH could contribute to the regulation of its own operon. For this, we engineered a strain where most of the coding region of *ydcH* was replaced by a spectinomycin resistance cassette (*ydcH*::*spc*; ABS1381) and combined this deletion with each of the four previously described reporters. We observed a strong induction of P_*ydc1*_*lacZ* and P_*ydc1-2*_*lacZ* fusions, while the expression of P_*ydc2*_ and P_*ydc0*_ remained identical to that observed in the wild type strain (Fig 2A). This indicates that YdcH has a repressing effect on the transcription of P_*ydc1*_, while P_*ydc2*_ remains unaffected by the presence or absence of YdcH. We concluded that, because the frameshift mutation observed in strain 3725 resulted in the truncation of half of YdcH, it is highly probable that this mutation was responsible for the induction of the operon in this strain. Since our strain deleted for *mbl* was previously entirely sequenced and showed no additional mutations, this in turn explains the discrepancy of expression between *mreB* and *mbl* mutants regarding the expression of the *ydc* locus (Fig 1B) [40].

Finally, we tested if the two other genes of the operon could also contribute to the regulation of their own expression. To avoid any polar effect on *ydcH*, we generated marker-less inactivation mutants of *ydcF* and *ydcG* by eviction of the erythromycin resistant cassette in, respectively, the *ydcF*::*ery* (BKE4750) and *ydcG*::*ery* (BKE4760) clones from a *B*. *subtilis* deletion library ([41]; see Materials and methods for details). However, no impact on the expression of either P_*ydc1*_ (ASEC297; ASEC301) or P_*ydc2*_ (ASEC333; ASEC335) could be detected in these mutants (Fig 2A).

In order to get better insight into the regulated P_*ydc1*_ promoter, we turned to a more sensitive and dynamic approach than the β-galactosidase reporter, and constructed a transcriptional fusion between P_*ydc1*_ and *luxABCDE* encoding the bacterial luciferase from *Photorhabdus luminescence* instead of *lacZ* (ABS2005; see Methods). When *B*. *subtilis* cells are grown in rich LB medium, they grow exponentially for 90 minutes, before being subject to a slow down (Fig 2B), or transition phase, for an additional 80 minutes until they enter a final phase marked by a very slow growth, or stationary phase (Fig 2B). While our β-galactosidase reporter indicated no expression originating from P_*ydc1*_ in a wild type context (Fig 2A), we could observe with the luciferase reporter that P_*ydc1*_ is in fact transiently expressed for about 1.5 h. Its expression was just above detection levels and peaked at the transition from exponential to stationary phase (Fig 2B). A similar transient induction was observed when cells were grown in the rich CH or minimal CE and S media (S1 Fig).

Thanks to the sensitivity of the luciferase reporter, we were able to observe, depending on the medium, a 3 to 9 and 5 to 12 fold increase of expression in the absence of *ydcF* and *ydcG*, respectively, compared to the wild type levels (Fig 2B and S1 Fig). Albeit significant, the increase was two orders of magnitude lower that that observed in absence of *ydcH* (ASEC329; Fig 2B and S1 Fig), matching our observations with the β-galactosidase reporter. This indicates that repression was still largely maintained in the absence of YdcF or YdcG suggesting only a minor role, if any, of these proteins on the repression of P_*ydc1*_. Taken together, our observations indicate that expression of the *ydcFGH* operon occurs during exponential growth, but is maintained to very low levels in an YdcH dependent manner.

### *ydcH* binds to the promoter of the *ydcFGH* operon

YdcH is predicted to belong to the MarR family of TR. These proteins are homodimers with a characteristic winged-Helix-Turn-Helix (wHTH) domain conferring affinity for 16–20 base pair (bp) inverted repeats [7]. Most MarR family members act as repressors, by binding close to, or overlapping, the -35 or -10 sequences of their target promoters, hence preventing the binding of the RNA polymerase through steric hindrance [6].

**Fig 3:**
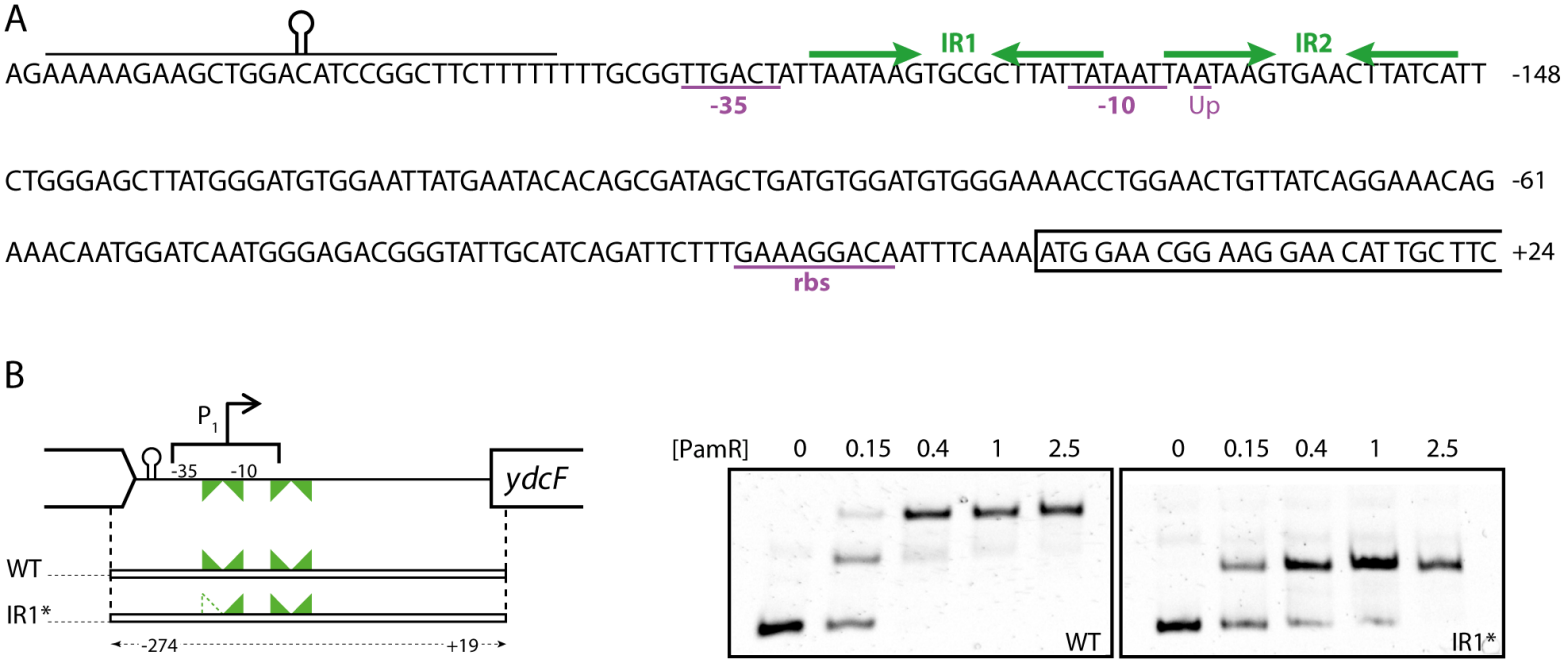
YdcH binds specifically to inverted repeats in the promoter region of *ydcFGH*. A. Sequence of the region upstream of the *ydcFGH* operon. The two identified IR are indicated as green arrows. The transcriptional upshift previously identified is indicated as “up”, putative -35, -10 and rbs sequences are underlined, and the *ydcF* orf is boxed. B. EMSAs (right panels) showing the specific binding of PamR to DNA fragments corresponding to the wild type (wt) and mutated (IR1*) *ydcF* promoter, and schematic representation of the corresponding area (left panel). IRs are drawn as facing triangles, plain for the wild types and hollowed for the mutated. The quantity of YdcH (in pmol) incubated with 0.1 pmol of labeled target DNA is indicated above each lane.

To test if the repressing effect of YdcH on P_*ydc1*_ expression that we observed could be due to the direct binding of the protein to the promoter, we purified a recombinant form of the protein and tested its binding potential in an electrophoretic mobility shift assay (EMSA). Although the promoter was not mapped, whole genome tailing arrays [39] had shown a transcriptional upshift 165 bp upstream of the translational start site of the *ydcF* orf. Analysis of this area revealed directly upstream of this upshift the presence of -35 and -10 sequences typical of σA-dependent promoters and, in between, a perfect inverted repeat (IR) sequence (TAATAAGnnnnCTTATTA) forming a palindrome (Fig 3A). We first incubated a 293 bp DNA fragment extending from the end of the upstream *rsbX* gene to the beginning of the *ydcF* orf with purified YdcH, in the presence of an excess of non-specific competitor DNA (Fig 3B). We observed specific, concentration-dependent retardations, suggesting a direct and specific binding of the protein (Fig 3B; wt). Interestingly three species appeared indicating the formation of two DNA-protein complexes.

The complete retardation was observed with a ratio of 4 moles of proteins per mole of DNA, consistent with the binding of 2 dimers per DNA molecules. A rapid examination of the promoter sequence revealed the presence of a second imperfect direct repeat (TAATAAGnnnnCTTATcA) overlapping the probable transcriptional initiating base (Fig 3A; IR2), that could account for the observed complexes. To test if the directed repeats correspond to the motif recognized by YdcH, we created a DNA fragment mutant for the most upstream IR, by swapping between both strands of the seven bases forming the first half of the IR (ATTATTCnnnnCTTATTA). The resulting EMSA with the mutated DNA fragment showed that one of the protein-DNA interactions was abolished, indicating that the IRs are the probable motifs recognized by YdcH. We then scanned the entire *B*. *subtilis* genome for the two identified IR but these sequences were not found anywhere else.

### *ydcFGH* is not involved in resistance to a variety of stresses

We next wondered what could be the function of this operon. *ydcF* is predicted to encode a 97 amino-acid peptide with no similarity to known or uncharacterized proteins. The only noticeable characteristic of YdcG is the presence of a domain of unknown function, EVE (formerly DUF055), structurally related to the RNA-binding PUA domain, present in all three kingdoms of life with a predominance in bacteria [42]. Thus, no clear function could be infered from these two genes. However, our results are in full agreement with YdcH acting as a repressor. MarR TRs are usually involved in the response to environmental changes, helping cells to improve their survival, and are frequently linked to multiple resistance to antibiotic, salt and aromatic molecules and to virulence [6, 43–45]. We therefore evaluated the effect of deleting each of the *ydcFGH* genes on the ability of *B*. *subtilis* to resist to various stresses.

**Fig 4:**
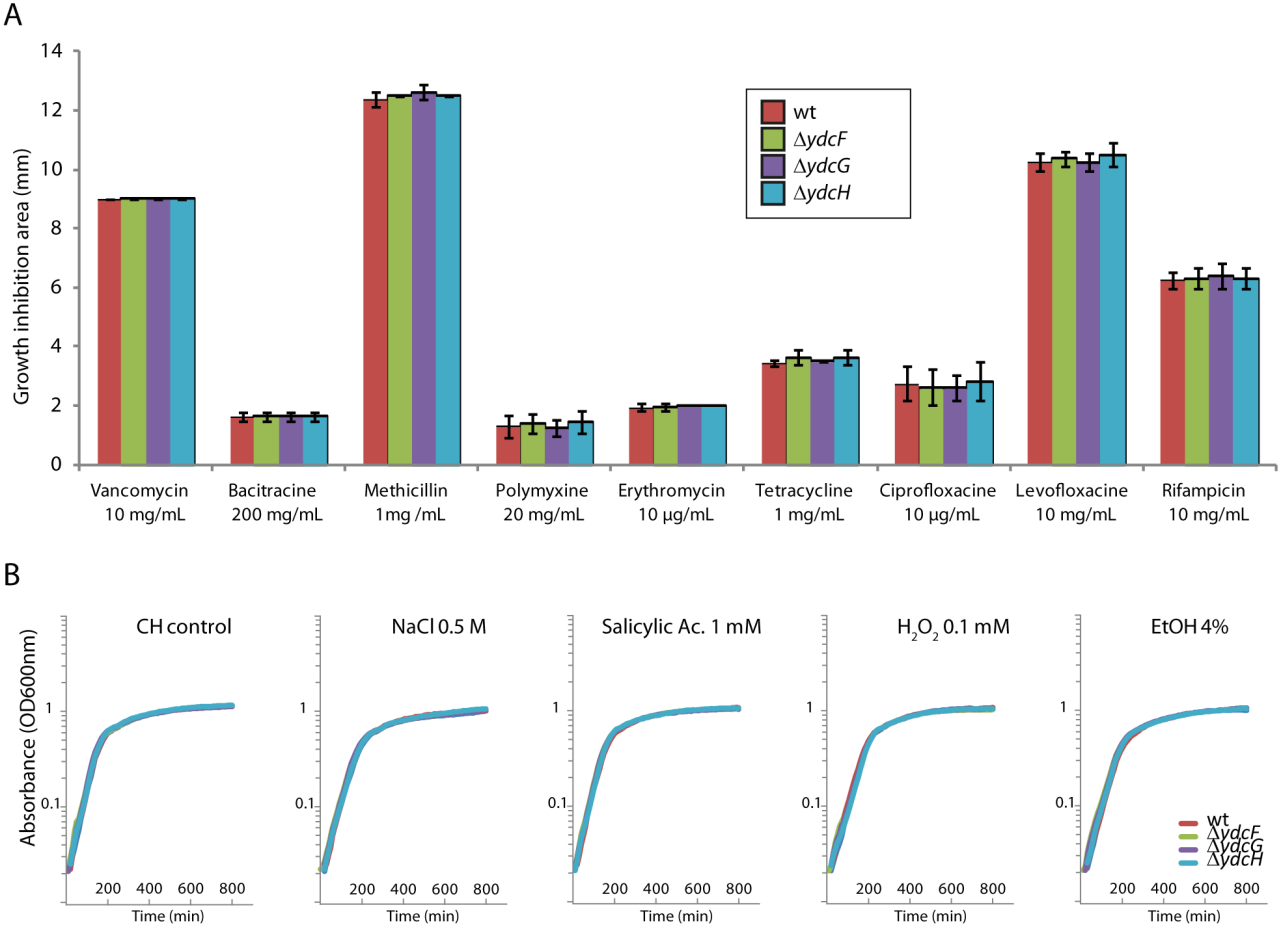
YdcF, YdcG and YdcH are not involved in a variety of stress resistances. A. Growth inhibition areas induced by antibiotics in disc diffusion assay, between Wt (168) and mutant strains for *ydcF* (AECS287), *ydcG* (ASEC289) and *ydcH* (ABS1381). Antibiotics tested and their respective initial concentration when spotted, are indicated on the graphic. B. Growth curves in rich CH medium of Wt (ABS2005) and mutant strains for *ydcF* (ASEC325), *ydcG* (ASEC327) and *ydcH* (ASEC329) in response to NaCl (0.5M), Salicylic acid (1mM), H_2_0_2_ (0.1mM) and ethanol (4%).

Using disc diffusion assays, we tested the resistance of the three deletion mutants to antibiotics of various classes, affecting cell wall synthesis (Vancomycin, Bacitracine, Methicillin), membrane integrity (polymyxine), replication (ciprofloxacine, levofloxacine) transcription (rifampicine) or translation (erythromycin, tetracycline) (Fig 4A), but no benefits or impairments could be observed in these mutants relative to the WT strain. Nicolas and coworkers systematic expression profiling study showed an induction of the operon in response to the presence of ethanol, high osmolarity and oxidative stress [39]. Thus, we next tested the impact of high concentration of NaCl, Ethanol, and H_2_O_2_, on the survival of all three mutants, together with Salicylic acid, a frequent inducer of MarR-type TR (Fig 4B), but no significant differences could be observed. It should be noted that no significant induction of our P_*ydc1*_ *luxABCDE* fusion could be detected in any of these conditions, in the wild type as in the mutants (S2 Fig).

### PamR, a new global regulator acting on carbon metabolism and prophages control

Our results strongly suggest that YdcH is a transcriptional repressor of the MarR family of TF. Although we could not detect elsewhere in the genome the exact direct repeats identified in the promoter region of *ydcF*, we could not rule out the possibility that YdcH could recognize degenerated motifs. We reasoned that the deletion of *ydcH* should inform us on putative direct targets with degenerated binding sequences or indirect targets affected by the upregulation of the *ydc* operon that could lead us to the potential biological function of this operon. We thus conducted a whole genome transcription profiling by RNAseq, comparing differential gene expression between Δ*ydcH* (ASEC329) and its parental strain, wild type for *ydcH* (ASB2005). Samples of control and mutant strains were collected during mid-exponential growth in rich CH medium and RNA were extracted, sequenced and analyzed as described in the methods section.

**Fig 5:**
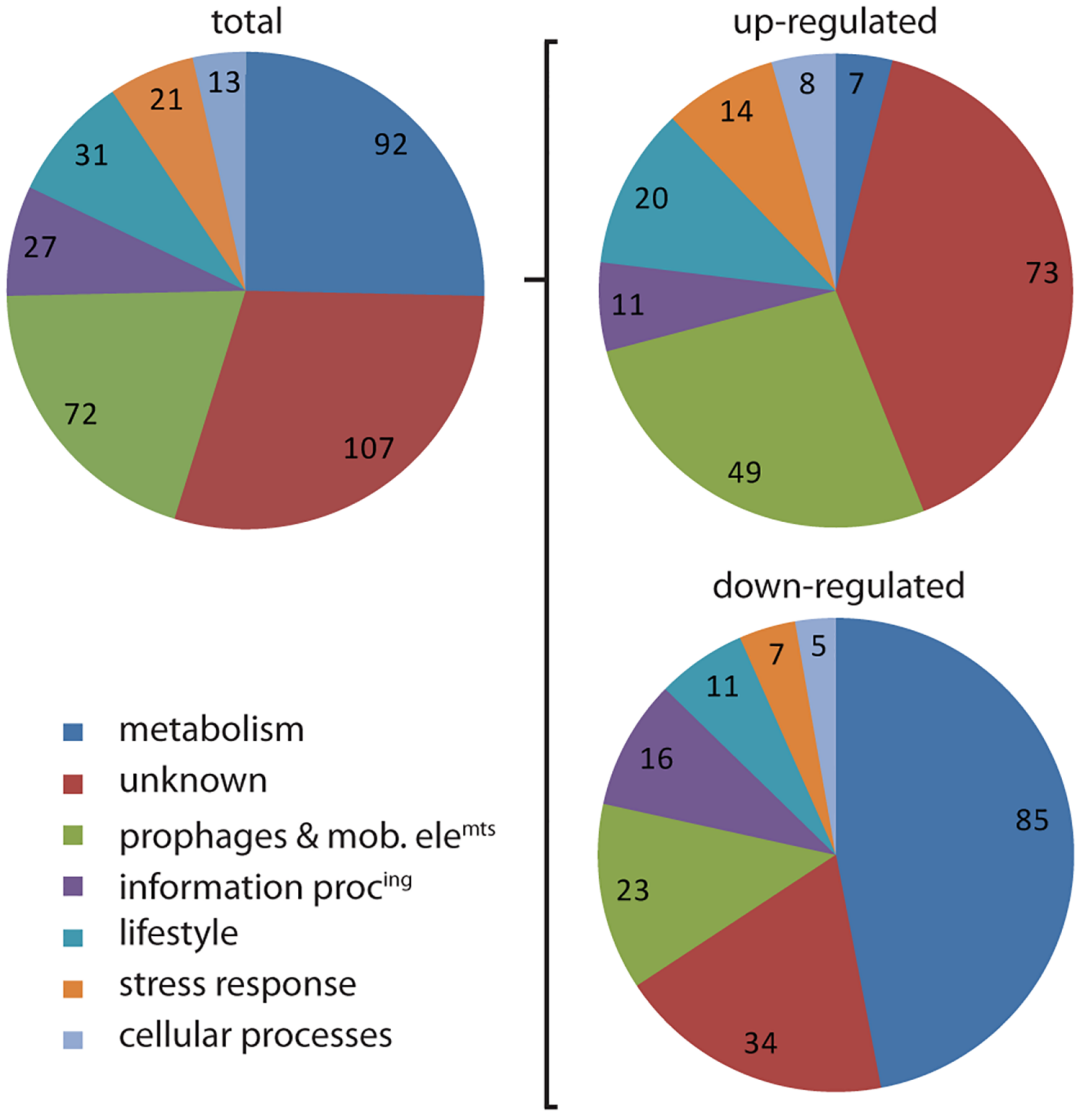
The YdcH regulon. Pie charts summarizing genome-wide transcriptional profiling by RNAseq comparing gene expressions in a WT (ABS2005) and a Δ*ydcH* strain (ASEC56). The 363 genes retained (left chart) were reproducibly and statistically induced (182, right up) or repressed (181, right down) in the mutant compared to the wt by at least a two-fold factor. Genes were sorted by functional categories (see S5 Table for complete results), then regrouped into families of functions: Metabolism (carbon sources, amino acids, lipids, nucleotides and other metabolic pathways; electron transport & ATP synthesis; transport of sugars and other metabolites), stress response, information processing (DNA replication, segmentation, modification, recombination and repair; RNA and protein synthesis, modification and degradation), cellular processes (cell division; cell envelope synthesis, modification and degradation; ion homeostasis), lifestyles (motility & chemotaxis; biofilms formation; competence; sporulation), prophages & mobile genetic elements, and unknown. Numbers indicate the number of gene for each category.

For our analysis, we retained only the transcripts that statistically significantly varied between the two strains across three independent replicates, and above a threshold of 2 fold of induction/repression. A substantial number of 363 genes were affected in the Δ*ydcH* mutant compared to the wt strain and were almost evenly distributed between up- and down-regulated genes (182 and 181 genes, respectively) (Fig 5 and S5 Table). Among them, we noticed a large fraction of genes of unknown function. This is not surprising considering that there is still over 800 proteins of unknown function (~20% of the total number of protein) to date in *B*. *subtilis* according to the Subtiwiki database (http://www.subtiwiki.uni-goettingen.de/). Interestingly, the remaining genes present a clear bias for two functional categories: i- metabolism-associated functions, including intermediate metabolic enzymes, sugar transporters, and metabolic regulators, and ii- prophages and other mobile genetic elements. Most genes involved in metabolism were down regulated in the *ydcH* mutant, as well as genes belonging to the PBSX prophage, while genes belonging to all others prophage or mobile genetic element (SPβ prophage, skin element, Prophage1 and ICEBs1) were induced in absence of *ydcH* (Fig 5 and S5 Table). It is noticeable that 40 genes encoding TF or other regulators (including 16 known transcription factors) (Table 2), acting either on metabolic pathways (LrpA, LrpB, ThrR, TnrA, NtdR, PyrR), control of prophages (ImmR, ImmA, Xre, SknR, RapI) or during transition phase (AbrB, SinR) and stationary phase events such as sporulation (SigF, SpoVT, Sda, YisI) or competence (ComK, Kre) were also affected. These regulators reflect well the diversity of functional categories identified in our transcriptomic analysis by RNAseq (Fig 5) and may also account for the large number of genes whose expression differ in our mutant. In agreement, among the genes affected by *ydcH* absence we noticed that 34 fall under AbrB (a transition state TR) regulation, a majority of them (25/34) variying as if AbrB was less active in the Δ*ydcH* strain (AbrB-induced genes are down and AbrB-repressed are up). We also noticed that 42 known targets of CcpA, a transcriptional repressor of catabolic genes, are down regulated as if CcpA was more active in the Δ*ydcH* mutant. Since expression of *ccpA* did not seem affected in absence of *ydcH*, it suggests that different levels of regulations could be affected in this mutant.

**Table 2:** Transcription factors and other regulators affected in the absence of PamR.

gene	Effect^3^	Exp. Diff.	TR & Mod ^1^	Functional category ^2^	Function
*ypoP*	+	3,00	TF*	U	similar to transcriptional regulator (MarR family)
*yopO*	+	4,12	TF*	Prophages & mobile gen. elem^ts^	similar to transcriptional regulator (Xre family)
*yonR*	+	5,29	TF*	Prophages & mobile gen. elem^ts^	similar to transcriptional regulator (Xre family)
*yobD*	+	3,69	TF*	U	similar to transcriptional regulator (Xre family)
*ykvN*	+	2,64	TF*	U	unknown; MarR family transcription regulator
*ydfL*	+	3,30	TF*	U	unknown; similar to multidrux efflux transporter regulator of MreR family
*ydeB*	+	3,26	TF*	U	unknown; putative transcriptional regulator
*yceK*	+	2,56	TF*	U	unknown; putative transcriptional regulator
*ybzH*	+	3,84	TF*	Prophages & mobile gen. elem^ts^	unknown; putative transcriptional regulator
*xre*	+	3,83	TF	Prophages & mobile gen. elem^ts^	regulation of PBSX prophage gene expression
*tnrA*	+	2,45	TF	Metabolism	control of nitrogen assimilation (MerR family)
*sknR*	+	3,04	TF	Prophages & mobile gen. elem^ts^	repression of *yqaF* operon of the skin element (Xre family)
*sinR*	+	2,76	TF	Lifestyle	regulator of post-exponential-phase responses genes, and biofilm formation
*ntdR*	+	3,73	TF	Lifestyle	activator of *ntdABC-glcP* operon (LacI family)
*mntR*	+	2,77	TF	Cellular processes	regulation of manganese transport (DtxR family)
*mgsR*	+	2,86	TF	Stress response	regulator of a subset of the SigB stress regulon; oxidative stress protection
*lrpB*	+	3,51	TF	Metabolism	metabolism of glycine; Repression of glyA transcription and KinB-dependent spo.
*lrpA*	+	5,26	TF	Metabolism	metabolism of glycine; Repression of glyA transcription and KinB-dependent spo.
*immR*	+	3,86	TF	Prophages & mobile gen. elem^ts^	Control of transfer of the mobile genetic element ICEBs1
*comK*	+	2,74	TF	Lifestyle	master regulator for competence
*abrB*	+	4,34	TF	Lifestyle	tansition state regulator
*sigX*	+	3,07	TF	Stress response	resistance to cationic antimicrobial peptides; RNA polymerase ECF-type σ factor
*pyrR*	+	3,56	R	Metabolism	transcriptional antiterminator of the pyr operon
*kre*	+	3,31	R	Lifestyle	inhibitor of competence; ComK repressor
*yydG*	+	4,50	R	Stress response	control of LiaR-LiaS activity; oxidoreductase
*yisI*	+	4,16	R	Lifestyle	inhibition of phosphorelay; Spo0A-P phosphatase
*sda*	+	2,68	R	Lifestyle	checkpoint coupling replication and sporulation; inhibitor of Spo0A phosphorelay
*rapI*	+	2,96	R	Prophages & mobile gen. elem^ts^	control of ICEBs1, ImmR antagonist; response regulator aspartate phosphatase
*phrE*	+	3,48	R	Lifestyle	Regulator of RapE phosphatase
*immA*	+	3,40	R	Prophages & mobile gen. elem^ts^	control of ImmR activity; site-specific protease
*degR*	+	3,58	R	Metabolism	control of DegU; Positive effector of DegU-phosphate stability
*yhcF*	-	3,26	TF*	U	unknown; putative transcription factor (GntR family)
*thrR*	-	2,75	TF	Metabolism	control of threonine biosynthesis
*spoVT*	-	2,37	TF	Lifestyle	regulation of forespore gene expression
*sigF*	-	2,56	TF	Lifestyle	RNA polymerase forespore-specific (early) sigma factor SigF
*ycbM*	-	2,71	R*	U	predicted two-component sensor kinase
*yhcY*	-	2,13	R	U	two component sensor kinase
*yesM*	-	4,38	R	U	two-component sensor kinase
*psdS*	-	2,41	R	Stress response	two-component sensor kinase, response to lipid II-binding lantibiotics
*crh*	-	2,43	R	Metabolism	CcpA cofactor

^1^ "TF" stands for transcriptional factor,

* indicates its function is putative, based on sequence similarity,

R stands for other Regulatory function

^2^ "U" stands for unknown

^3^ +/- impact of Δ*pamR* on expression of the gene

Taken together, these results indicate that, albeit potentially indirect through activity of other regulators, the absence of *ydcH* leads to a global reprogramming of the cell with a specific prominence of metabolic pathways and prophage-related genes. We therefore propose to rename the gene *pamR* for prophages and metabolism control regulator.

## Discussion

Our study originally focused on the *ydcFGH* operon because of its potential relationship with MreB, aiming at deciphering both its function and the link with this morphoprotein. However our results indicate that induction of this operon was genetically unlinked with the morphogene. Because mutants of *mreB* are notoriously sick and easily accumulate suppressor mutations in the absence of high concentrations of Mg^2+^ [37, 46, 47], we cannot exclude that the presence of the mutation affecting *pamR* (*ydcH*) in *mreB* 3725 strain is the result of a selective pressure on this strain. Puzzlingly, we also noticed in the literature that a strain selected for L-form proficiency (PDC134) (i.e. a strain supporting growth without cell wall) was reported to bear 16 intragenic mutations (28 mutations in total), all of them present in the 3725 strain [38]. The common origin of strain PDC134 and 3725 suggests that they share a common ancestor presenting these mutations rather than having been selected independently. In addition, we could not observe any benefit of the deletion of *pamR* when inserted in a Δ*mreB* mutant, on the growth or shape of this mutant. Because the genome of strain 3725 contains 50 other sequence variations relative to its wild type parental strain, it is therefore possible that one of the other mutations induced a change that in turn required the inactivation of *pamR*.

But what could be the function of the *ydc* operon? Our results have yet to demonstrate a function for the first two genes of the operon. However, we showed that PamR (YdcH) controls its own expression, specifically binds (probably as a dimer) its own promoter due to the presence of two IRs motif (TAATAAGtgnnCTTATNA) overlapping the RNA polymerase binding site, strongly suggesting that it is indeed a transcriptional regulator acting as a repressor by preventing the recruitment of the RNA polymerase by steric hindrance. This is a frequent feature of MarR TRs that often control their expression by binding to the promoter controlling their own gene or operon [6–8]. Using genome wide transcription profiling, we also showed that the absence of PamR correlates with the induction or repression of numerous genes, leading to a reprogramming of the *B*. *subtilis* genetic expression larger in its extent than the general stress response (namely the SigB regulon [48, 49]), the SigH-dependent stationary phase adaptation [14] and similar to stationary phase developmental processes as competence or sporulation [50, 51]. TRs usually bind DNA with a strong affinity for a specific palindromic sequence located in the promoter region of the controlled genes and inhibiting transcription by steric hindrance or more rarely inducing transcription [7, 8]. While most MarR-type TRs generally act as repressors and control small regulons, PamR uncommonly affects a very large number of genes, some positively and some negatively in rather equal proportions. Although it is at this stage possible that PamR is an atypical MarR-type TR, it is more likely that the vast majority of affected genes, if not all but the *ydcFGH* operon, are downstream consequences of expression changes affecting its “core” regulon, i.e. genes under direct PamR control. This is re-enforced by the absence of conserved PamR binding site in the rest of the genome. A clue advocating for this is the fact that many regulators or modulators of regulators are affected by *pamR* deletion along with at least a fraction of their respective regulons (Xre, AbrB….). Thus by affecting the expression or/and activity of a limited number of key proteins, it would allow a global reprogramming of the cell. What could be the purpose of such regulation? The pattern of expression of the P_*ydc1*_ promoter (controlling the three genes of the *ydcFGH* operon) shows induction during the transition between exponential and stationary phase. Although this may be due to an additional regulatory process, it may nevertheless suggest a function in the transition between growth phases, a hypothesis reinforced by several observations. First, there is an overlap between the PamR and AbrB regulons, AbrB being a well-known transition state regulator [13]. In absence of PamR, we observed a release of the repression of a part of the AbrB regulon suggesting a partial decrease in AbrB repression capacity. Thus PamR may contribute to the previously reported sequential AbrB de-repression observed from mid-exponential growth to stationary phase [52]. Next, we observed a change of expression of a large group (92) of metabolic genes that could reflect a growth adaptation and a need to cope with new carbon sources. We also observed the presence of numerous transition or stationary phase-involved genes including antibiotic/toxin producing operons (*albA*, *skf*, *yydG*…) that suggest a scavenging strategy, and stress response genes that also advocate for adaptation to a complex life style and/or stationary phase. Finally, the induction of genes from the lysogenic prophages SPβ and the mobile elements ICEBs1 could also indicate a response to stress. DNA damages are the most documented cause promoting their activation [20, 21]. It is however unlikely that the SOS response could here be responsible for the induction of SPβ and ICEBs1 because only a very limited number of LexA-controlled genes (the repressor controlling the SOS response) are induced (and some are even down-regulated) in absence of PamR. In addition, the defective prophage PBSX, also known to be induced in response to DNA damage [53], is repressed in this genetic context. Other stresses have been shown to promote their activation including reactive oxygen species, heat, some antibiotics and toxic compounds [24] but the absence of induction of the general stress response in absence of PamR advocate against this possibility. Together this suggests that a more complex regulatory network could be responsible for the differential expression of these prophage-encoded genes.

However, considering the very mild induction observed with our reporter (in the wt background) in every conditions tested so far compared to its levels in the strain deleted for *pamR*, it is probable that in our conditions most of the *pamR* regulon will barely be affected. Uncovering the conditions of optimal activation of the PamR regulon would help discovering the signal sensed by the regulator. Typically, MarR-type TRs bind to one or several ligands upon which the affinity to DNA decreases, allowing the release of repression–or activation- to their target genes. From our results, the first two genes of the operon (*ydcF* and *ydcG*) are not -or marginally- involved in P_*ydc1*_ regulation suggesting they are not involved in the production or sensing of the yet to be determined signal.

Overall, based on the extent of the regulon and the observed influence on stationary-phase, stress-response, metabolic or phage-related genes, our results suggest that PamR is switching the genetic program of the bacteria in response to an unknown signal to help the cell cope with some environmental changes or growth regime modification.

## Supporting information

S1 Fig P_*ydc1*_ is strongly induced in the absence of YdcH in rich and poor media. Expression of a P_*ydc1*_ *luxABCDE* transcriptional fusion in cells grown in poor S and CE or rich CH media, in a wild type (red; ABS2005) or mutant for *ydcF* (green; ASEC325), *ydcG* (purple; ASEC327) or *ydcH* (blue; ASEC329) background. Left panels do not include Δ*ydcH* (ASEC329) data that would be off chart (notice the different axis ranges between right and left panels). Growth curves are shown as dotted lines and correspond to the optical density at 600nm while luciferase activities (plain lines) are relative luminescence units normalized by the OD600nm.
(PDF)

S2 Fig P_*ydc1*_ is not induced in response to various stresses. Maximum expression of a P_*ydc1*_ luxABCDE transcriptional fusion in cells exposed to NaCl (0.5 M), Salicylic acid (1 mM), H_2_0_2_ (0.1 mM) and ethanol (4%), in a wild type (ABS2005) or mutant for *ydcF* (ASEC325), *ydcG* (ASEC327) or *ydcH* (ASEC329) background.
(PDF)

S1 Table Strains used in this study. (PDF)

S2 Table Plasmids used in this study. (PDF)

S3 Table Oligonucleotides used in this study. (PDF)

S4 Table Sequence variations detected in strain 3725 (detailed). (PDF)

S5 Table List of differentially expressed genes in a Δ*pamR* strain compared to its parental wild type *B*. *subtilis* strain. (PDF)
