# Supplementary figures and images for: PamR, a new MarR-like regulator affecting prophages and metabolic genes expression in Bacillus subtilis
Source: PLoS One. 2017 Dec 14;12(12):e0189694. doi: 10.1371/journal.pone.0189694 (PMC5730154; doi:10.1371/journal.pone.0189694)

Fig S1 de San Eustaquio et al.

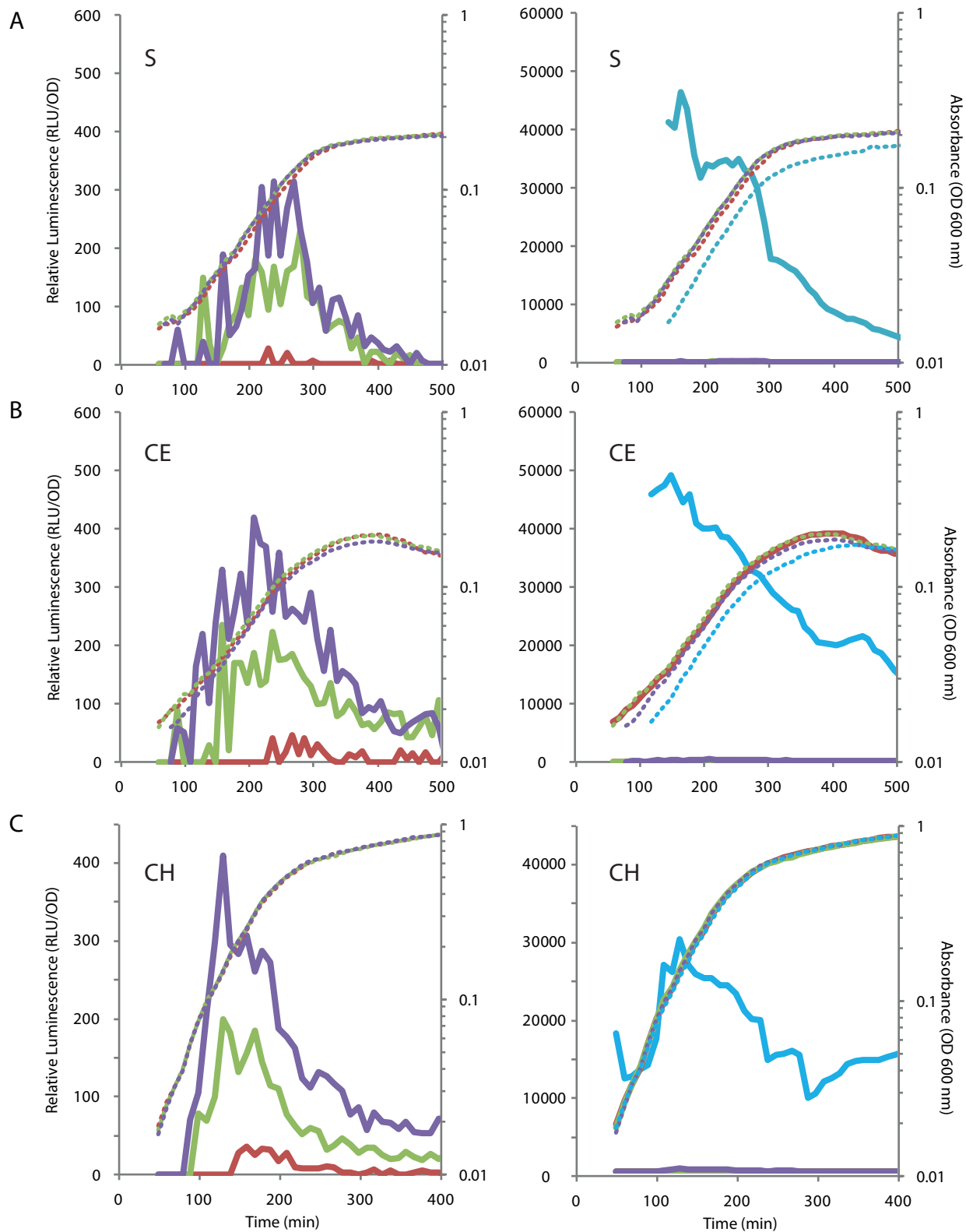

Supplement: S1 Fig — Expression of a Pydc1 luxABCDE transcriptional fusion in cells grown in poor S and CE or rich CH media, in a wild type (red; ABS2005) or mutant for ydcF (green; ASEC325), ydcG (purple; ASEC327) or ydcH (blue; ASEC329) background. Left panels do not include ΔydcH (ASEC329) data that would be off chart (notice the different axis ranges between right and left panels). Growth curves are shown as dotted lines and correspond to the optical density at 600nm while luciferase activities (plain lines) are relative luminescence units normalized by the OD600nm. (PDF) [file pone.0189694.s001.pdf]

Fig S2 de San Eustaquio et al.

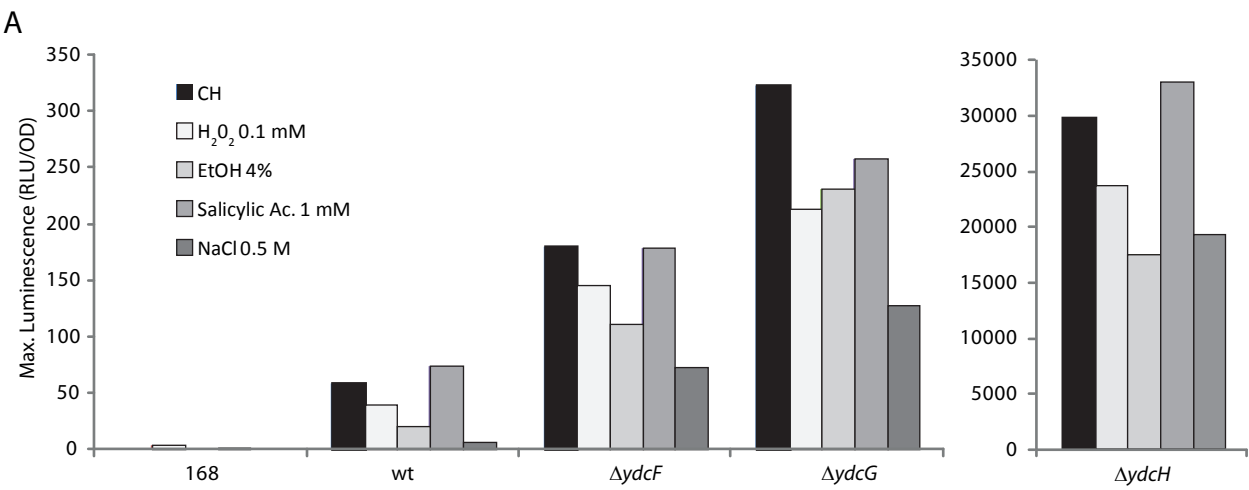

Supplement: S2 Fig — Maximum expression of a Pydc1 luxABCDE transcriptional fusion in cells exposed to NaCl (0.5 M), Salicylic acid (1 mM), H202 (0.1 mM) and ethanol (4%), in a wild type (ABS2005) or mutant for ydcF (ASEC325), ydcG (ASEC327) or ydcH (ASEC329) background. (PDF) [file pone.0189694.s002.pdf]
