# Supplementary material for: PamR, a new MarR-like regulator affecting prophages and metabolic genes expression in Bacillus subtilis
Source: PLoS One. 2017 Dec 14;12(12):e0189694. doi: 10.1371/journal.pone.0189694 (PMC5730154; doi:10.1371/journal.pone.0189694)
Supplement: S1 Table — (PDF) [file pone.0189694.s003.pdf]

TABLE S1. Strains used in this study

name	relevant genotype	construction or reference
E. coli:		
DH5a	*F- endA1 glnV44 thi-1 recA1 relA1 gyrA96 deoR nupG Φ80dlacZΔM15 Δ(lacZYA-argF)U169, hsdR17(rK- mK+), λ–*	stock collection
BL21	*F- λ- fhuA2 [lon] ompT lacZ::T7 gal sulA11 Δ(mcrC-mrr)114::IS10 R(mcr-73::miniTn10-TetS)2 R(zgb-210::Tn10)(TetS) endA1 [dcm]*	stock collection
EC.ASEC19	pET(P_{T7} *his-ydcH, lacI, kan*)	pET_ydcH_H-Nt ->DH5a
B. subtilis:		
168	wild type "168-Oxford" (24 variations with GenBank AL009126.3)	stock collection
RCL78	Δ*mbl* ::*cm*	(1)
3725	neo-Δ*mreB*	(2)
ABS1761	*amyE* ::P_{ydc1} *lacZ-spc*	pAC769 -> 168
ABS1763	*amyE* ::P_{ydc2} *lacZ-spc*	pAC772 -> 168
ABS1765	*amyE* ::P_{ydc1-2} *lacZ-spc*	pAC775 -> 168
ABS1767	*amyE* ::P_{ydc0} *lacZ-spc*	pAC778 -> 168
ABS1762	*amyE* ::P_{ydc1} *lacZ-spc ; neo-* Δ*mreB*	pAC769 -> 3725
ABS1769	*amyE* ::P_{ydc1} *lacZ-spc ;* Δ*mbl* ::*cm*	RCL78 -> ABS1761
ABS1381	Δ*ydcH* ::*spc*	LFH PCR -> 168
ABS1798	Δ*ydcH* ::*spc* ::*erm*	pQP1 -> ABS1381
ABS1820	Δ*ydcH* ::*spc* ::*erm ; amyE::*P_{ydc1} *lacZ-spc*	pAC769 -> ABS1798
ABS1821	Δ*ydcH* ::*spc* ::*erm ; amyE::*P_{ydc2} *lacZ-spc*	pAC772 -> ABS1798
ABS1822	Δ*ydcH* ::*spc* ::*erm ; amyE::*P_{ydc1-2} *lacZ-spc*	pAC775 -> ABS1798
ABS1823	Δ*ydcH* ::*spc* ::*erm ; amyE::*P_{ydc0} *lacZ-spc*	pAC778 -> ABS1798
BKE04750	Δ*ydcF* ::*erm*	BKE collection (BGSC)
BKE04760	Δ*ydcG* ::*erm*	BKE collection (BGSC)
BKE04770	Δ*ydcH* ::*erm*	BKE collection (BGSC)
ASEC275	Δ*ydcF* ::*erm*	BKE4750 -> 168
ASEC277	Δ*ydcG* ::*erm*	BKE4760 -> 168
ASEC279	Δ*ydcH* ::*erm*	BKE4770 -> 168
ASEC287	Δ*ydcF**	pDR244 -> ASEC275
ASEC289	Δ*ydcG**	pDR244 -> ASEC277
ASEC333	Δ*ydcF* * ; *amyE* ::P_{ydc2} *lacZ-spc*	ABS1763 -> ASEC287
ASEC335	Δ*ydcG* * ; *amyE* ::P_{ydc2} *lacZ-spc*	ABS1763 -> ASEC289
ABS2057	*sacA* ::*Km*	pSac-Kan -> 168
ABS2084	*sacA* ::*km* ::P_{ydc1} *lacZ-cm*	pAC826 -> ABS2057
ASEC281	Δ*ydcF* ::*erm ; sacA* ::*km* ::P_{ydc1} *lacZ-cm*	BKE04750 -> ABS2084
ASEC283	Δ*ydcG* ::*erm ; sacA* ::*km* ::P_{ydc1} *lacZ-cm*	BKE04760 -> ABS2084
ASEC297	Δ*ydcF* * ; *sacA* ::*km* ::P_{ydc1} *lacZ-cm*	pDR244 -> ASEC281
ASEC301	Δ*ydcG* * ; *sacA* ::*km* ::P_{ydc1} *lacZ-cm*	pDR244 -> ASEC283
ABS2005	*sacA* ::P_{ydc1s} *luxABCDE-cm*	pAC834 -> 168
ASEC311	Δ*ydcF* ::*erm ; sacA* ::P_{ydc1s} *luxABCDE-cm*	ASEC275 -> ABS2005
ASEC313	Δ*ydcG* ::*erm ; sacA* ::P_{ydc1s} *luxABCDE-cm*	ASEC277 -> ABS2005
ASEC315	Δ*ydcH* ::*erm ; sacA* ::P_{ydc1s} *luxABCDE-cm*	ASEC279 -> ABS2005
ASEC325	Δ*ydcF* * ; *sacA* ::P_{ydc1s} *luxABCDE-cm*	pDR244 -> ASEC311
ASEC327	Δ*ydcG* * ; *sacA* ::P_{ydc1s} *luxABCDE-cm*	pDR244 -> ASEC313
ASEC329	Δ*ydcH* * ; *sacA* ::P_{ydc1s} *luxABCDE-cm*	pDR244 -> ASEC315
ASEC56	Δ*ydcH* ::*spc ; sacA* ::P_{ydc1s} *luxABCDE-cm*	ABS2005 -> ABS1381

* stands for markerless deletion
