# Supplementary material for: PamR, a new MarR-like regulator affecting prophages and metabolic genes expression in Bacillus subtilis
Source: PLoS One. 2017 Dec 14;12(12):e0189694. doi: 10.1371/journal.pone.0189694 (PMC5730154; doi:10.1371/journal.pone.0189694)
Supplement: S2 Table — (PDF) [file pone.0189694.s004.pdf]

TABLE S2. Plasmids used in this study

Plamsid name	relevant information	construction or reference
pET28a	vector for protein expression with a T7 IPTG-inducible promoter	Novagen
pET_ydcH_6H-Nt	vector for Histidine-tagged YdcH expression and purification	PCR(acec64-4) -> pET28a
pDR244	vector with ts ori, expressing cre recombinase allowing excition of *erm* cassette in BKE strains	(3)
pQP1	plasmid allowing deletion/replacement of the *spc* resistance casette by *ery*	laboratory stock
pSac-Kan	plasmid allowing introduction of a kanamycin cassette at the *sacA* locus	(4)
pSac-Cm	plasmid allowing introduction of a chloramphenicol cassette at the *sacA* locus	(4)
pDG1728	suicide vector for integration of transcriptional fusions to *lacZ* (and *spc*) at the *amyE* locus	(5)
pDG1663	suicide vector for integration of transcriptional fusions to *lacZ* (and *erm*) at the *thrC* locus	(5)
pAH328	suicide vector for integration of transcriptional fusions to *luxABCDE* (and *cm*) at the *sacA* locus	(6)
pAC769	pDG1728 derivative for integration of P_{ydc1} *lacZ* transcriptional fusion at *amyE*	PCR(ac1240-41) -> pDG1728
pAC772	pDG1728 derivative for integration of P_{ydc2} *lacZ* transcriptional fusion at *amyE*	PCR(ac1243-42) -> pDG1728
pAC775	pDG1728 derivative for integration of $P_{ydc1\text{-}2}$ *lacZ* transcriptional fusion at *amyE*	PCR(ac1240-42) -> pDG1728
pAC778	pDG1728 derivative for integration of P_{ydc0} *lacZ* transcriptional fusion at *amyE*	PCR(ac1243-41) -> pDG1728
pAC826	pSac-Cm derivative for integration of P_{ydc1} *lacZ* transcriptional fusion at *sacA*	PCR(ac1240-80) -> pSac-Cm
pAC834	pAH328 derivative for integration of P_{ydc1s} *luxABCDE* transcriptional fusion at *sacA*	PCR(ac1240-87) -> pAH328
