# Supplementary material for: PamR, a new MarR-like regulator affecting prophages and metabolic genes expression in Bacillus subtilis
Source: PLoS One. 2017 Dec 14;12(12):e0189694. doi: 10.1371/journal.pone.0189694 (PMC5730154; doi:10.1371/journal.pone.0189694)
Supplement: S3 Table — (PDF) [file pone.0189694.s005.pdf]

## TABLE S3. Oligonucleotides used in this study

| name | sequence (5' to 3') | usage |
|---|---|---|
| ac-1240 | GTAGAATTCGCTGAAAATGTATACGACATCGAG | cloning $P_1$ or $P_{1+2}$ in front of *lacZ* |
| ac-1241 | GGAGGATCCCCGTCGGCATGTCTTTAGACAGT | cloning $P_1$ or $P_0$ in front of *lacZ* |
| ac-1242 | GGAGGATCCGGCAGCGCCTTTTAATACATGTT | cloning $P_2$ or $P_{1+2}$ in front of *lacZ* |
| ac-1243 | GAAGAATTCACTTATCATTCTGGGAGCTTATGGG | cloning $P_2$ or $P_0$ in front of *lacZ* |
| ac-1280 | ACAAAGCTTAAAAACTGGTCTGATCGATAG | cloning $P_1$ in front of *lacZ* at *sacA* |
| ac-1287 | ACTACTAGTAATCTGATGCAATACCCGTC | cloning $P_1$ in front of *lux* at *sacA* |
| ac-1052 | TCGGCGTTGTGTCAGAGCAACAT | LFH-PCR of *ydcH* ::*spc* (replacement) |
| ac-1053 | CAATTCGCCCTATAGTGAGTCGTGGCTTGTGTTCGGACCGCTAAA | " |
| ac-1054 | CCAGCTTTTGTTCCCTTTAGTGAGTGAGCGGCTTACAGGAACTATTGA | " |
| ac-1055 | TTGCTTTTGGCGACTGTCGCCTT | " |
| ac-1056 | GTGGGGAGAAATCATTGGGACTG | *ydcH* locus sequencing |
| ac-1057 | GAACACTTCTTTTTCTTCGCTG | " |
| asec-04 | GGA GGA TCC CCC GTA TCT TTA CTC ATG TT | cloning ydcH in pET28a |
| asec-64 | CCACCATGGTCCATCATCATCATCATCATGGCGGTGTGGAATGGCACTCTAAATTTAGCGGTC | " |
| ac-1385 | AATGTTCCTTCCGTTCC | EMSA, mutagenesis (reverse-dn) |
| ac-1386 | GCGCAGAATAATATAGTCAACCGCAAA | EMSA, mutagenesis (reverse-up) |
| ac-1387 | TGCGGTTGACTATATTATTCTGCGCT | EMSA, mutagenesis (forward-dn) |
| ac-1390 | GATGAAAAACAAAAGAGGCG | EMSA, mutagenesis (forward-up) |
| ac-1391 | ACCTGACCTATATTCTCGG | EMSA, Cy-5 labelled |
| ac-1392 | AATGTTCCTTCCGTTCC | " |

**References:**


1. Billaudeau C, Chastanet A, Yao Z, Cornilleau C, Mirouze N, Fromion V, et al. Contrasting mechanisms of growth in two model rod-shaped bacteria. Nature communications. 2017;8:15370.
2. Formstone A, Errington J. A magnesium-dependent *mreB* null mutant: implications for the role of *mreB* in *Bacillus subtilis* . Molecular microbiology. 2005;55(6):1646-57.
3. Wang X, Montero Llopis P, Rudner DZ. *Bacillus subtilis* chromosome organization oscillates between two distinct patterns. Proceedings of the National Academy of Sciences of the United States of America. 2014;111(35):12877-82.
4. Middleton R, Hofmeister A. New shuttle vectors for ectopic insertion of genes into *Bacillus subtilis* . Plasmid. 2004;51(3):238-45.
5. Guerout-Fleury AM, Frandsen N, Stragier P. Plasmids for ectopic integration in *Bacillus subtilis* . Gene. 1996;180(1-2):57-61.
6. Chen Y, Cao S, Chai Y, Clardy J, Kolter R, Guo JH, et al. A *Bacillus subtilis* sensor kinase involved in triggering biofilm formation on the roots of tomato plants. Molecular microbiology. 2012;85(3):418-30.
