# Supplementary material for: PamR, a new MarR-like regulator affecting prophages and metabolic genes expression in Bacillus subtilis
Source: PLoS One. 2017 Dec 14;12(12):e0189694. doi: 10.1371/journal.pone.0189694 (PMC5730154; doi:10.1371/journal.pone.0189694)
Supplement: S4 Table — (PDF) [file pone.0189694.s006.pdf]

Table S4. Sequence variations detected in strain 3725

gene[1]	position[2]	variation type	change[3] Ref.	Obs.	aminoacid	function	functional category		essential
**Common variations to wild type and mutant strains (relative to the published 168 sequence)**									
**INTRAGENIC**									
uvrX	2271523	Non Synonymous Coding	A	C	D45E	UV DNA damage repair	DNA repair	information processing	no
rrnI-16S	161239	untranslated region	T	TC	NA	ribosomal RNA 16S	translation	information processing	no
trnI-asn	165825	untranslated region	G	GC	NA	transfer RNA-asn	translation	information processing	no
rrnG-16S	171679	untranslated region	TA	T	NA	ribosomal RNA 16S	translation	information processing	no
rrnB-16S	3178443	untranslated region	T	C	NA	ribosomal RNA 16S	translation	information processing	no
pstS (5'UTR)	2581726	untranslated region	G	GT	NA	phosphate ABC tranporter	Phosphate metabolism	Metabolism	no
ydzN (5'UTR)	557865	untranslated region	G	GT	NA	unknown	unknown	unknown	
yjpA (3'UTR)	1317152	untranslated region	G	GGT	NA	unknown	unknown	unknown	no
yjpA (3'UTR)	1317153	untranslated region	C	CT	NA	unknown	unknown	unknown	no
zwf (ter)	2480646	untranslated region	T	A	NA	glucose 6P desyhdrogenase	carbon metabolism	Metabolism	no
zwf (ter)	2480647	untranslated region	A	T	NA	glucose 6P desyhdrogenase	carbon metabolism	Metabolism	no
zwf (ter)	2480653	untranslated region	CT	C	NA	glucose 6P desyhdrogenase	carbon metabolism	Metabolism	no
zwf (ter)	2480666	untranslated region	GT	G	NA	glucose 6P desyhdrogenase	carbon metabolism	Metabolism	no
ywbD (ter)	3935822	untranslated region	AT	A	NA	unknown	unknown	unknown	no
**synonymous codon (silent)**									
uvrX	2271424	Synonymous coding	T	C	R78	UV DNA damage repair	DNA repair	information processing	no
uvrX	2271505	Synonymous coding	C	T	V51	UV DNA damage repair	DNA repair	information processing	no
**INTERGENIC**									
int (up *trnI-asn*)	165748	untranslated region	TC	T	NA	transfer RNA-asn	translation	information processing	no
int (up *trnI-asn*)	165750	untranslated region	TC	T	NA	transfer RNA-asn	translation	information processing	no
int (dn *trnI-gly*)	166037	untranslated region	A	AT	NA	transfer RNA-gly	translation	information processing	no
int (dn *trnI-ala*)	166343	untranslated region	AG	A	NA	transfer RNA-ala	translation	information processing	no
int (*s1555* /*purA*)	4155390	untranslated region	C	CA	NA	tRNA-lys/adenylosuccinate synthetase	translation/purine acquisition	information processing/Metabolism	no
int (*yocK* /*azoR1*)	2097080	untranslated region	C	CA	NA	ethanol response/azoreductase	stress response/stress response	Lifestyle/Lifestyle	no
s1417	3770058	untranslated region	G	GAAA[illegible]	NA	unknown	unknown	unknown	no
int (*ydgF* /*dinB*)	608214	untranslated region	G	GA	NA	unknown/nuclease inhibitor	unknown/DNA repair	unknown/inhibitor processing	no
**Variations in Δ*mreB* 3725 relative to its parental wild type 168 strain**									
**Variations in strain 3725 also reported in strain PDC134**									
**INTRAGENIC**									
sepF	1610896	Non Synonymous Coding	T	C	M11T	recruits cell division FtsZ protein to membrane	Cell division	cellular process	no
walR	4153797	Non Synonymous Coding	G	A	R203C	two component response regulator	CW homeostasis	cellular process	YES
ytpS (=*sftA*)	3051461	Non Synonymous Coding	G	A	P375S	DNA translocase (paralog of SpoIIIE)	DNA segregation	information processing	no
epsC	3527377	Non Synonymous Coding	G	A	A276V	udp-sugar epimerase (inactive in 168)	biofilm	Lifestyle	no
comP	3253956	Non Synonymous Coding	T	C	E628G	two component sensor kinase, snsing ComX	competence and sporulation	Lifestyle	no
sigI	1412484	Non Synonymous Coding	T	G	L198R	alternative sigma factor	stress response	Lifestyle	no
bscR (=*FatR*)	2777176	Non Synonymous Coding	A	C	I160R	Transcriptional repressor of *fatR-yrhJ* operon	Fatty acid metabolism	Metabolism	no
ymfD	1756603	Non Synonymous Coding	G	A	A319T	bacillibactin exporter	iron acquisition	Metabolism	no
mmgA	2513699	Non Synonymous Coding	A	C	M115R	degradative acetoacetyl-CoA thiolase	Leucine metabolism	Metabolism	no
yuxG (=*rahEW*)	3202213	Non Synonymous Coding	C	T	G573R	bifunctional rhamnulose-P aldolase/lactate deshydrogenase	Rhamnose catabolism	Metabolism	no
ydcH	525386	indel: Frame Shift	CA	C	frame shift	unknown	unknown	unknown	no
yhgE	1091083	Non Synonymous Coding	G	A	G228E	unknown	unknown	unknown	no
yisQ & *S389*	1161993	Non Synonymous Coding	C	T	D51N	unknown	unknown	unknown	no
yjcM & *S430*	1264284	Non Synonymous Coding	T	A	K216N	unknown	unknown	unknown	no
BSU_misc_RNA_20	1424639	untranslated region	T	G	NA	unknown; coexpressed with MtnK (methionine salvage)	unknown	unknown	no
panE	1514277	Non Synonymous Coding	G	A	A76T	unknown; putative ketopentoate reductase	unknown	unknown	no
BSU_misc_RNA_28	1620435	untranslated region	GT	G	NA	unknown	unknown	unknown	no
yxbD	4095811	Non Synonymous Coding	C	T	V9I	unknown	unknown	unknown	no
**INTERGENIC**									
s1123 (5'utr *NifZ)* /*s1124* (5'utr *braB*)	3028250	untranslated region	A	T	NA	cystein desulfurase/branched-chain aa transporter	tRNA modification/metabolism	information processing/metabolism	no
s1145 (5' utr *ytnP*)	3059481	untranslated region	AC	A	NA	lactonase homologue	antibactrial compound	lifestyle	no
s125 (5' utr *hxlR*)	376017	untranslated region	AG	A	NA	Transcriptional regulator (formaldehyde detoxification)	stress resistance	lifestyle	no
s486 (5' UTR *mtnE*)	1425560	untranslated region	C	A	NA	glutamine transaminase	methionine salvage	Metabolism	no
int *yorN* /*yorM*	2174751	untranslated region	ACT	A	NA	SPbeta prophage	prophage	prophage & Mobile genetic elements	no
s352 (3' Utr *yhaH*)	1073872	untranslated region	AT	A	NA	unknown	unknown	unknown	no
s736 (dnstream *yocJ*)	2096245	untranslated region	TA	T	NA	unknown	unknown	unknown	no
s596 (5'utr *ylxY*)	1741603	untranslated region	AG	A	NA	unknown; similar to deacetylase	unknown	unknown	no

**Variations unique to strain 3725**									
**INTRAGENIC**									
parC	1936310	Non Synonymous Coding	C	T	S288L	subunit of DNA topoisomerase IV	DNA segregation	information processing	YES
rRNA23S	34740	untranslated region	A	G	NA	ribosomal RNA 23S	translation	information processing	no
ylyB	1618068	Non Synonymous Coding	C	T	P287S	unknown, similar to pseudourilydate synthase	translation	information processing	no
trmD	1675849	Non Synonymous Coding	C	T	H227Y	tRNA methyltransferase	translation	information processing	YES
pksN	1841168	indel: Frame Shift	C	CA	frame shift	polyketide synthase, type I	antibactrial compound synthesis	Lifestyle	no
oppD	1224524	Non Synonymous Coding	T	G	V357G	oligopeptide ABC transporter	competence and sporulation	Lifestyle	no
gerAA	3391676	Non Synonymous Coding	A	G	T299A	nutrient receptor (L-Ala) promoting spore germination	germination	Lifestyle	no
s1255 /sspG	3354116	indel: codon deletions	G(8)*	G	8 codons deleted	sspG: small acidic soluble protein/S1255: unknown	sporulation	Lifestyle	no
accC	2530964	Non Synonymous Coding	T	C	D248G	acetyl-coA carboxylase	fatty acid biosynthesis	Metabolism	YES
glcK	2571163	indel: Frame Shift	G	GT	frame shift	glucose kinase	glucose metabolism	Metabolism	no
gltA	2011091	Non Synonymous Coding	G	A	A1181V	glutamate synthase	metabolism	Metabolism	no
sacA	3902306	Non Synonymous Coding	C	A	L448F	phosphosucrase	sucrose catabolism	Metabolism	no
ilvC & *s1070*	2893906	Non Synonymous Coding	G	A	A268	ketol-ac reductoisomerase/unknown	aminoacids synthesis/unknown	Metabolism/unknown	no
yoqA	2201408	Non Synonymous Coding	A	G	L23P	unknown; SPβ prophage	prophage	Prophage & Mobile genetic elements	no
yqbS	2670611	Non Synonymous Coding	A	G	Y60H	unknown; skin element	prophage	Prophage & Mobile genetic elements	no
yesY	774697	indel: Frame Shift	CA	C	frame shift	unknown, similar to rhamnogalacturonan acetylesterase	unknown	unknown	no
yutE	3319154	Non Synonymous Coding	C	T	A37T	unknown	unknown	unknown	no
**synonymous codon (silent)**									
pgdS	3696869	Synonymous coding	T	C	L210	gamma-DL-glutamyl hydrolase	capsule biosynthesis	cellular process	no
rluB	2421606	Synonymous coding	T	C	Q189	pseudouridine synthase	translation	information processing	no
yqeZ	2619105	Synonymous coding	C	T	A225	Ser protease, resistance to sublancin	stress response	Lifestyle	no
ygaN	965353	Synonymous coding	A	T	S31	unknown	unknown	unknown	no
yozT	2041099	Synonymous coding	A	G	D43	unknown	unknown	unknown	no
**INTERGENIC**									
int *bsrB* /*yrvM*	2814468	untranslated region	C	T	NA	bsrB: sigA antognist / yrvM: cyclase	gene regulation/ modification	information processing	no
s1101 (5' utr *CitZ*)	2982417	untranslated region	T	A	NA	citrate synthase (TCA cycle)	metabo	Metabolism	no
s1101 (5' utr *CitZ*)	2982437	untranslated region	T	C	NA	citrate synthase (TCA cycle)	metabo	Metabolism	no

[1] "int" stands for intergenic region, "utr" for untranslated region

[2] nucleotide position relative to the *B. subtilis* 168 sequence from GenBank (AL009126.3)

[3] "Ref." and "Obs." stand for nucleotide(s) present in the reference and the 3725 sequences, respectively

*G(GCAAAAGTCCAAAACAGCGGTAAT)
