# Supplementary material for: PamR, a new MarR-like regulator affecting prophages and metabolic genes expression in Bacillus subtilis
Source: PLoS One. 2017 Dec 14;12(12):e0189694. doi: 10.1371/journal.pone.0189694 (PMC5730154; doi:10.1371/journal.pone.0189694)
Supplement: S5 Table — (PDF) [file pone.0189694.s007.pdf]

Table S5. List of differentially expressed genes in a Δ*pamR* strain compared to its parental wild type *B. subtilis* strain

gene	Effect of Δ*pamR*	Exp. Diff.	Functional category	Function	Regulated by			
**genes repressed in absence of *pamR* during exponential growth**								
rasP	-	2,67	Cellular processes_Cell division	intramembrane protease; control of cell division, SigV and SigW activity (via FtsL, RsiV and RsiW clivage)	U			
gtcA	-	2,21	Cellular processes_Cell wall synthesis and homeostasis	teichoic acid glycosylation protein	CcpA (-)			
cpgA	-	2,29	Cellular processes_Cell wall synthesis and homeostasis	GTPase	U			
prpC	-	2,95	Cellular processes_Cell wall synthesis and homeostasis	protein phosphatases_PrkC antagonist	U			
murF	-	2,22	Cellular processes_Cell wall synthesis and homeostasis_biosynthesis of peptidoglycan	peptidoglycan precursor biosynthesis	SigM	SigV		
mutSB	-	2,17	Information processing_DNA replication, repair & recombination	probable DNA mismatch repair protein	U			
sbcE	-	2,30	Information processing_DNA replication, repair & recombination	DNA double-strand break repair and competence; SMC-like protein	LexA (-)			
dnaI	-	2,34	Information processing_DNA replication, repair & recombination	primosome component (helicase loader)	U			
parE	-	2,55	Information processing_DNA replication, repair & recombination	chromosome segregation_subunit of DNA topoisomerase IV	LexA (-)			
mutM/ytaE	-	3,04	Information processing_DNA replication, repair & recombination	formamidopyrimidine-DNA glycosidase	SigE			
gltX	-	2,09	Information processing_Protein synthesis	translation; glutamyl-tRNA synthetase	T-box			
fmt/yloL	-	2,34	Information processing_Protein synthesis	tRNA modification; methionyl-tRNA formyltransferase	SigA			
engA	-	2,41	Information processing_Protein synthesis	ribosome assembly ; essential GTPase for ribosome 50S assembly	U			
queA	-	2,45	Information processing_Protein synthesis	tRNA modification; S-adenosylmethionine tRNA ribosyltransferase	U			
valS	-	2,58	Information processing_Protein synthesis	translation; valyl-tRNA synthetase	T-box			
cca	-	2,63	Information processing_Protein synthesis	tRNA modification; tRNA nucleotidyltransferase	Spx			
pheS	-	2,70	Information processing_Protein synthesis	translation; phenylalanyl-tRNA synthetase (alpha subunit)	T-box			
trmK	-	2,74	Information processing_Protein synthesis	tRNA modification; tRNA:m1A22 methyl transferase	U			
truA/ybaH	-	2,88	Information processing_Protein synthesis	tRNA modification; pseudouridylate synthase I	RelA (-)			
yukB	-	2,39	Information processing_Protein synthesis_secretion	part of the type VII protein secretion system; ATPase	DegU (+)			
deaD	-	2,16	Information processing_RNA synthesis and degradation	DEAD-box RNA helicases	U			
yxaB	-	2,85	Lifestyle_biofilm formation	putative exopolysaccaride synthase	SigB	AbrB (-)		
acpK	-	14,40	Lifestyle_Biosynthesis of antibacterial compounds	polyketide biosynthesis, acyl carrier protein	AbrB (-)	CodY (+)		
cheR	-	2,30	Lifestyle_Motility & chemotaxis	MCPs methyltransferase	U			
motA	-	2,36	Lifestyle_Motility & chemotaxis	H+-coupled flagellar stator	SigD			
flhG	-	2,59	Lifestyle_Motility & chemotaxis	GTPase activating protein, activates FlhF	SigD	CodY	DegU	Spo0A, SwrAA/1
spoVT	-	2,37	Lifestyle_Sporulation	regulation of forespore gene expression; transcription regulator	SigF/G			
sigF	-	2,56	Lifestyle_Sporulation	RNA polymerase forespore-specific (early) sigma factor SigF	AbrB (-)	Spo0A (+)	SinR (-)	SigF/G
yhbB/ygaQ	-	2,67	Lifestyle_Sporulation	spore coat protein, amidase	SigE			
ytaF	-	2,72	Lifestyle_Sporulation	U	SigE			
thiQ	-	3,14	Lifestyle_Sporulation	U	SigE	SpoIIID (-)		
tepA	-	2,49	Lifestyle_Sporulation_germination	degradation of SASPs; orphan ClpP-like germination protease	SigG	SpoVT (-)		
aroF	-	2,34	Metabolism_Biosynthesis & acquisition of Aminoacids	chorismate synthase	sigA			
tyrA	-	2,50	Metabolism_Biosynthesis & acquisition of Aminoacids	biosynthesis of tyrosine; prephenate dehydrogenase	MtrB	TRAP (-)		
proB	-	2,53	Metabolism_Biosynthesis & acquisition of Aminoacids	biosynthesis of proline; glutamate 5-kinase	T-box			
trpD	-	2,66	Metabolism_Biosynthesis & acquisition of Aminoacids	biosynthesis of tryptophan, anthranilate phosphoribosyltransferase	MtrB	TRAP (-)		
artQ	-	2,72	Metabolism_Biosynthesis & acquisition of Aminoacids	Arginine uptake; arginine ABC transporter (permease)	casa ac (-)			
aroB	-	2,74	Metabolism_Biosynthesis & acquisition of Aminoacids	3-dehydroquinate synthase	sigA			
thrR	-	2,75	Metabolism_Biosynthesis & acquisition of Aminoacids	TR, control of threonine biosynthesis	U			
hisH	-	2,93	Metabolism_Biosynthesis & acquisition of Aminoacids	histidine biosynthesis; imidazole glycerol phosphate synthase (glutaminase subunit)	U			
hisA	-	3,07	Metabolism_Biosynthesis & acquisition of Aminoacids	biosynthesis of histidine; phosphoribosylformimino-5-aminoimidazole carboxamide ribotide isomerase	U			
trpF	-	3,26	Metabolism_Biosynthesis & acquisition of Aminoacids	synthesis of tryptophan, phosphoribosylanthranilate isomerase	MtrB	TRAP (-)		
hisB	-	3,70	Metabolism_Biosynthesis & acquisition of Aminoacids	biosynthesis of histidine; imidazoleglycerol-phosphate dehydratase	U			
mccB	-	4,43	Metabolism_Biosynthesis & acquisition of Aminoacids	biosynthesis of cystein; methionine-to-cysteine conversion; cystathionine lyase/ homocysteine lyase	CymR (-)	Spx (+)		
mccA	-	4,51	Metabolism_Biosynthesis & acquisition of Aminoacids	biosynthesis of cystein; methionine-to-cysteine conversion; O-acetylserine-thiol-lyase	CymR (-)	Spx (+)		
lplJ	-	2,14	Metabolism_Biosynthesis of cofactors	Lipoate:protein ligase	U			
moaE	-	2,29	Metabolism_Biosynthesis of cofactors	nitrate respiration; molybdopterin synthase (large subunit)	U			
panB	-	2,40	Metabolism_Biosynthesis of cofactors	biosynthesis of coenzyme A; 3-methyl-2-oxobutanoate hydroxymethyltransferase	U			
pdxK	-	2,49	Metabolism_Biosynthesis of cofactors	pyridoxal biosynthesis; kinase	U			
birA	-	2,50	Metabolism_Biosynthesis of cofactors	Transcriptional regulator; Biotin-protein ligase	BirA (-)	Spx (+)		
bioW	-	2,86	Metabolism_Biosynthesis of cofactors	Biotin synthesis; pimeloyl-CoA synthase	BirA (-)			
thiU	-	2,88	Metabolism_Biosynthesis of cofactors	thiamine transporter (binding protein)	Thi-box			
folC	-	2,91	Metabolism_Biosynthesis of cofactors	biosynthesis of folate ; folyl-polyglutamate synthetase	T-box			
thiS	-	3,43	Metabolism_Biosynthesis of cofactors	biosynthesis of thiamine; sulfur carrier protein	Thi-box			
bioF	-	3,70	Metabolism_Biosynthesis of cofactors	Biotin synthesis; 8-amino-7-oxononanoate synthase	BirA (-)			
tenI	-	3,97	Metabolism_Biosynthesis of cofactors	biosynthesis of thiamine; thiazole tautomerase	Thi-box			
sumT	-	9,87	Metabolism_Biosynthesis of cofactors	siroheme biosynthesis , sulfite reduction;probable uroporphyrin-III C-methyltransferase	CymR (-)	S-box		
ykuN	-	2,54	Metabolism_Electron transport & ATP synthesis	electron transfer; flavodoxin	Fur (-)	NsrR (-)	Kre (-)	ResD (+)
atpF	-	2,56	Metabolism_Electron transport & ATP synthesis	ATP synthase(Fo complex subunit b)	RelA (-)			
atpH	-	2,96	Metabolism_Electron transport & ATP synthesis	ATP synthase(Fo complex subunit d)	RelA (-)			
etfB	-	3,22	Metabolism_Electron transport & ATP synthesis	fatty acid degradation, electron transfer flavoprotein (beta subunit)	CcpA (-)	FadR (-)		

resB/ypxB	-	2,36	Metabolism_Electron transport & ATP synthesis_Respiration	cytochrome c biogenesis; part of the ResB-ResC haem translocase	CcpA (-)	PhoP (+)	ResD (+)		
pfkA	-	2,69	Metabolism_Glycolysis	6-phosphofructokinase	Glucose (+)				
dhbB	-	2,24	Metabolism_iron metabolism	isochorismatase; biosynthesis of the siderophore bacillibactin	Fur (-)	AbrB (-)	Kre (-)		
sufU	-	2,58	Metabolism_iron metabolism	biosynthesis of iron-sulfur clusters; Fe-S cluster scaffold protein	U				
mobB	-	2,66	Metabolism_Nitrate respiration	molybdopterin-guanine dinucleotide cofactor synthesis protein	U				
dra	-	2,24	Metabolism_nucelotide metabolism	nucleotide catabolism_deoxyribose-phosphate aldolase	CcpA (-)	DeoR (-)			
nupC	-	3,02	Metabolism_nucelotide metabolism	pyrimidine nucleoside transport protein	CcpA (-)	DeoR (-)			
purH	-	2,90	Metabolism_nucleotide metabolism	purine biosynthesis; phosphoribosylaminoimidazole carboxamide formyltransferase	PurR (-)				
pyrF	-	3,63	Metabolism_nucleotide metabolism	pyrimidine biosynthesis, orotidine-5-phosphate decarboxylase	PyrR				
araL	-	4,58	Metabolism_poorly characterized	sugar-P phosphatase	CcpA (-)	AraR (-)			
crh	-	2,43	Metabolism_regulation of carbon flux	CcpA cofactor	CcpA (-)				
rbsA	-	3,09	Metabolism_Sugar uptake	Ribose uptake; ABC transporter (ATP-binding protein)	CcpA (-)	AbrB (+)			
glcK	-	3,14	Metabolism_Sugar uptake	glucose kinase	U				
levG	-	4,59	Metabolism_Sugar uptake	fructose uptake; fructose-specific PTS, EIID component	CcpA (-)	LevR (+)	SigL		
rbsD	-	5,13	Metabolism_Sugar uptake	Ribose uptake; ABC transporter	CcpA (-)	AbrB (+)			
levD	-	6,11	Metabolism_Sugar uptake	fructose uptake; fructose-specific PTS, EIIA component	CcpA (-)	LevR (+)	SigL		
levF	-	7,25	Metabolism_Sugar uptake	fructose uptake; fructose-specific PTS, EIIC component	CcpA (-)	LevR (+)	SigL		
levE	-	7,43	Metabolism_Sugar uptake	fructose uptake; trigger enzyme:furctose PTS EIIB	CcpA (-)	LevR (+)	SigL		
cysH	-	2,67	Metabolism_sulfur metabolism	sulfate reduction, phosphoadenosine phosphosulfate sulfotransferase	CymR (-)	S-box			
sat	-	6,52	Metabolism_sulfur metabolism	sulfate activation, sulfate adenylyltransferase	CymR (-)	S-box			
cysC	-	7,16	Metabolism_sulfur metabolism	sulfate reduction and activation, adenylyl-sulfate kinase	CymR (-)				
citZ	-	2,38	Metabolism_TCA cycle	citrate synthase II	CcpA (-)	CcpC (-)			
mdh	-	2,47	Metabolism_TCA cycle	malate dehydrogenase	CcpA (-)	CcpC (-)			
sucC	-	2,58	Metabolism_TCA cycle	TCA cycle,succinyl-CoA synthetase (beta subunit)	CcpA (-)				
icd	-	2,64	Metabolism_TCA cycle	isocitrate dehydrogenase	CcpA (-)	CcpC (-)			
sdhA	-	2,78	Metabolism_TCA cycle	succinate dehydrogenase (flavoprotein subunit)	FsrA (-)				
sucD	-	2,91	Metabolism_TCA cycle	TCA cycle,succinyl-CoA synthetase (beta subunit)	CcpA (-)				
gcvT	-	2,41	Metabolism_utilization of amino acids	glycine utilization; aminomethyltransferase	Gly-box				
gcvPA	-	2,68	Metabolism_utilization of amino acids	glycine utilization; glycine decarboxylase (subunit 1)	Gly-box				
rocG	-	3,43	Metabolism_utilization of amino acids	arginine utilization; controls the activity of GltC; glutamate deshydrogenase	CcpA (-)	AbrB (-)	RocR (+)	AhrC (+)	SigL
bglA	-	2,35	Metabolism_utilization of beta-glucosides	utilization of beta-glucosides; 6-phospho-beta-glucosidase	U				
bglH	-	3,98	Metabolism_utilization of beta-glucosides	salicin utilization, phospho-beta-glucosidase	CcpA (-)	LicT			
fadF/ywjF	-	3,10	Metabolism_utilization of fatty acids	Fatty acid degradation; function unknown	FadR (-)				
galK	-	2,38	Metabolism_utilization of galactose	galactokinase	CcpA (-)				
iolE	-	3,11	Metabolism_utilization of inositol	myo-inositol catabolism, 2-keto-myo-inositol dehydratase, dehydration of 2-keto-myo-inositol	CcpA (-)	IolR (+)			
iolD	-	3,42	Metabolism_utilization of inositol	myo-inositol catabolism, formation of 5-deoxy-D-glucuronic acid (3rd reaction)	CcpA (-)	IolR (+)			
iolC	-	3,74	Metabolism_utilization of inositol	myo-inositol catabolism, formation of 2-deoxy-5-keto-gluconic acid-6-phosphate (5th reaction)	CcpA (-)	IolR (+)			
iolG	-	3,79	Metabolism_utilization of inositol	myo-inositol catabolism, inositol 2-dehydrogenase	CcpA (-)	IolR (+)			
iolB	-	4,64	Metabolism_utilization of inositol	myo-inositol catabolism; formation of 2-deoxy-5-keto-gluconic acid (4th reaction)	CcpA (-)	IolR (+)			
mtlD	-	3,49	Metabolism_utilization of mannitol	mannitol-1-phosphate 5-dehydrogenase	MltR				
mtlA	-	4,18	Metabolism_utilization of mannitol	mannitol uptake; trigger enzyme: mannitol-specific phosphotransferase system, EIICB of the PTS	MltR				
mtlF	-	6,38	Metabolism_utilization of mannitol	PTS mannitol-specific enzyme IIA component	MltR				
acuA	-	3,19	Metabolism_utilization of organic acids	control of AcsA activity; N-acetyltransferase	CcpA (-)				
citT	-	3,99	Metabolism_utilization of organic acids	two-component response regulator, regulation of citrate uptake	CitT				
malL	-	2,80	Metabolism_utilization of other Carbon sources	starch & maltodextrine utilization; alpha-glucosidase	U				
licB	-	4,30	Metabolism_utilization of other Carbon sources	lichenan uptake; trigger enzyme:lichenan PTS EIIB	CcpA (-)	LicR (+)			
mdxE	-	4,71	Metabolism_utilization of other Carbon sources	maltodextrin ABC transporter	U				
xynD	-	2,54	Metabolism_utilization of other polymeric carbohydrates	arabinoxylan degradation; arabinoxylan arabinofuranohydrolase	AbrB (-)				
gmuE/ydhR	-	2,52	Metabolism_utilization of specific C source	glucomannan utilization; mannose kinase	AbrB (+)	CcpA (-)	GmuR (-)		
gmuD/ydhP	-	2,53	Metabolism_utilization of specific C source	glucomannan utilization; phospho-beta-mannosidase	AbrB (+)	CcpA (-)	GmuR (-)		
gmuF/ydhS	-	2,86	Metabolism_utilization of specific C source	glucomannan utilization; mannose-6-P-isomerase	AbrB (+)	CcpA (-)	GmuR (-)		
gmuA/ydhN	-	2,91	Metabolism_utilization of specific C source	glucomannan-specific phosphotransferase system, EIIA component	AbrB (+)	CcpA (-)	GmuR (-)		
gmuC/ydhO	-	3,08	Metabolism_utilization of specific C source	glucomannan-specific phosphotransferase system, EIIC component	AbrB (+)	CcpA (-)	GmuR (-)		
kdgK	-	3,84	Metabolism_utilization of specific C source	utilization of galacturonic acid, 2-dehydro-3-deoxygluconokinase	CcpA (-)	KgdR (-)			
treA	-	2,98	Metabolism_utilization of trehalose	trehalose utilization_phospho-alpha-glucosidase	CcpA (-)	TreR (-)	PhoP (+)		
rhaM	-	3,68	Metabolsim_utilization of rhamnose	utilization of rhamnose, mutarotase	CcpA (-)	RhaR (-)			
xtmB	-	2,35	Prophages & mobile genetic elements_PBSX prophage	phage DNA replication; PBSX terminase	Xpf (+)				
xkdE	-	2,69	Prophages & mobile genetic elements_PBSX prophage	U	Xpf (+)				
xtmA	-	2,77	Prophages & mobile genetic elements_PBSX prophage	phage DNA replication; PBSX terminase	Xpf (+)				
xkdQ	-	2,78	Prophages & mobile genetic elements_PBSX prophage	U	U				
xtrA	-	2,83	Prophages & mobile genetic elements_PBSX prophage	U	Xre (-)				
xepA	-	2,86	Prophages & mobile genetic elements_PBSX prophage	phage release, PBSX prophage lytic exoenzyme	U				
xkdG	-	2,98	Prophages & mobile genetic elements_PBSX prophage	U	Xpf (+)				
xkdF	-	3,07	Prophages & mobile genetic elements_PBSX prophage	U	Xpf (+)				
xkdJ	-	3,08	Prophages & mobile genetic elements_PBSX prophage	U	Xpf (+)				
xkdH	-	3,12	Prophages & mobile genetic elements_PBSX prophage	U	Xpf (+)				
xhlA	-	3,13	Prophages & mobile genetic elements_PBSX prophage	host cell lysis upon induction of PBSX	U				
xkdS	-	3,13	Prophages & mobile genetic elements_PBSX prophage	U	U				
xkdI	-	3,14	Prophages & mobile genetic elements_PBSX prophage	U	Xpf (+)				

xkdX	-	3,21	Prophages & mobile genetic elements_PBSX prophage	U	U			
xkdR	-	3,30	Prophages & mobile genetic elements_PBSX prophage	U	U			
xkdC	-	3,43	Prophages & mobile genetic elements_PBSX prophage	U	Xre (-)			
xhlB	-	3,45	Prophages & mobile genetic elements_PBSX prophage	host cell lysis	U			
xkdW	-	3,64	Prophages & mobile genetic elements_PBSX prophage	U	U			
xkdK	-	3,65	Prophages & mobile genetic elements_PBSX prophage	U	Xpf (+)			
xkdB	-	3,77	Prophages & mobile genetic elements_PBSX prophage	U	Xre (-)			
xkdD	-	4,07	Prophages & mobile genetic elements_PBSX prophage	U	Xre (-)			
ykzM	-	4,73	Prophages & mobile genetic elements_PBSX prophage	U	U			
ykzK	-	5,55	Prophages & mobile genetic elements_PBSX prophage	U	U			
psdS	-	2,41	Stress response_Antibiotic resistance	two-component sensor kinase, response to lipid II-binding lantibiotics, such as nisin and gallidermin	PsdR			
yvlA	-	2,14	stress response_cell envelope stress resistance	U	SigW	AbrB (-)		
yknW	-	2,60	stress response_cell envelope stress resistance	modulates assembly of the YknX-YknY-YknZ ABC transporter for the export of the SdpC toxin	AbrB (-)	SigW		
yqfA	-	2,76	stress response_cell envelope stress resistance	flottilin-like protein, resistence against sublancin	SigW			
floT	-	2,44	Stress response_membrane dynamics	lipid microdomains organization; resistance to cefuroxime	SigW			
yuaF	-	2,59	Stress response_membrane dynamics	NfeD2, role in maintaining membrane integrity during conditions of cellular stress	SigW			
psdA	-	2,69	Stress response_resistance to antimicrobials	export of lantibiotics, such as nisin and gallidermin	PsdR (+)			
yhcY	-	2,13	U	two component sensor kinase	LiaR (+)			
ytlR	-	2,25	U	U	SigD			
yqeK	-	2,31	U	U	U			
yprA	-	2,33	U	putative helicase	U			
yloV	-	2,34	U	U	U			
ykuO	-	2,34	U	U	Fur (-)	NsrR (-)	Kre (-)	ResD (+)
yloM	-	2,36	U	similar to S-adenosyl-L-methionine-dependent 16S rRNA m5C967 methyltransferase	SigA			
yusY	-	2,37	U	similar to endopeptidase	U			
ypgQ	-	2,38	U	Mn2-dependent pyrophosphohydrolase	U			
yqjZ	-	2,42	U	U	U			
ycnE	-	2,42	U	U	U			
yyaE	-	2,43	U	similar to formate deshydrogenase, put electron transport	U			
yyzE	-	2,49	U	similar to PTS IIE	U			
ylmE	-	2,54	U	poorly charcterized; involved in control of the CoA pool	Spo0A (-)			
yloN	-	2,57	U	similar to rRNA methyltransferase	U			
ytpQ	-	2,59	U	maybe involved in Fe homeostasis	Spx (+)			
ycbM	-	2,71	U	predicted two-component sensor kinase	U			
ynbB	-	2,79	U	U	U			
ysmA	-	2,82	U	U	U			
yrhE	-	2,84	U	U	U			
yxiI	-	3,20	U	U	U			
yhcF	-	3,26	U	putative transcription factor (GntR family)	U			
lplA	-	3,44	U	putative ABC transporters	U			
yxiM	-	3,80	U	U	U			
yvrO	-	2,98	U (put based on regulation: Lifestyle_stationnary phase adaptation	putative ABC transporters	AbrB (-)	ComK (+)		
acuB	-	2,71	U (put based on regulation: metabolism)	U	CcpA (-)			
yjmB	-	2,86	U (put based on regulation: metabolism)	similar to symporter	CcpA (-)	ExuR (-)		
msmE	-	3,05	U (put based on regulation: metabolism)	putative melibiose uptake; ABC transporter	CcpA (-)			
lrgA	-	3,79	U (put based on regulation: metabolism)	putative pyruvate transporter	CcpA (-)	Kre (-)	LytT (+)	
yjmC	-	4,10	U (put based on regulation: metabolism)	may be involved in galacturonate utilization	CcpA (-)	ExuR (-)		
yesM	-	4,38	U (put based on regulation: metabolism)	two-component sensor kinase	CcpA (-)			
yxeK	-	7,19	U (put based on regulation: metabolism_sulfur metabo)	putative utilization of a modified cystein variant; similar to monoxigenase	CymR (-)			
yxeN	-	8,86	U (put based on regulation: metabolism_sulfur metabo)	putative cystein ABC transporter	CymR (-)			
yxeL	-	9,47	U (put based on regulation: metabolism_sulfur metabo)	putative sulfur N-acetlyase	CymR (-)			

genes induced in absence of *pamR* during exponential growth

ydjM	+	5,39	Cellular processes_Cell wall synthesis and homeostasis	May be involved in cell wall metabolism (WalR regulon)	WalR (+)	PhoP (+)
tagC	+	3,17	Cellular processes_Cell wall synthesis and homeostasis_Biosynthesis of teichoic acid	unknown function	LexA (-)	
tagD	+	3,17	Cellular processes_Cell wall synthesis and homeostasis_Biosynthesis of teichoic acid	Glycerol-3-phosphate cytidylyltransferase	WalR (+)	PhoP (-)
ggaB	+	3,31	Cellular processes_Cell wall synthesis and homeostasis_Biosynthesis of teichoic acid	Galactosamine-containing minor teichoic acid biosynthesis	SigA	
tagB	+	3,84	Cellular processes_Cell wall synthesis and homeostasis_Biosynthesis of teichoic acid	putative CDP-glycerol:glycerol phosphate glycerophosphotransferase	WalR (+)	PhoP (-)
tagA	+	4,24	Cellular processes_Cell wall synthesis and homeostasis_Biosynthesis of teichoic acid	UDP-N-acetyl-D-mannosamine transferase	WalR (+)	PhoP (-)
ggaA	+	4,64	Cellular processes_Cell wall synthesis and homeostasis_Biosynthesis of teichoic acid	Galactosamine-containing minor teichoic acid biosynthesis; acitivity unknown	SigA	
mntR	+	2,77	Cellular processes_Trace metal homeostasis (Cu, Zn, Ni, Mn, Mo)	Regulation of manganese transport; transcriptional regulator (DtxR family)	MntR	
hbs	+	2,92	Information processing_DNA condensation/segregation	DNA packaging; Non-specific DNA-binding protein	LexA (-)	SigH
secE	+	3,16	Information processing_Protein synthesis_secretion	Protein secretion; preprotein translocase	U	
rpmGC	+	2,83	Information processing_Protein synthesis_translation	Replaces L33 under conditions of zinc depletion; alternative ribosomal protein	Zur (-)	
rpsO	+	2,95	Information processing_Protein synthesis_translation	Ribosomal protein S15	U	
rpmB	+	3,45	Information processing_Protein synthesis_translation	Ribosomal protein L28	U	
rpmGB	+	3,49	Information processing_Protein synthesis_translation	Ribosomal protein L33b	U	
rpsT	+	3,81	Information processing_Protein synthesis_translation	Ribosomal protein S20	U	

trnD-Cys/trnR	+	2,97	Information processing_Protein synthesis_tRNA	tRNA-Cys	SigA			
trnD-Trp/trnR	+	3,16	Information processing_Protein synthesis_tRNA	tRNA-Trp	SigA			
trnD-Ser/trnR	+	3,44	Information processing_Protein synthesis_tRNA	tRNA-Ser	SigA			
trnB-Met3	+	3,62	Information processing_Protein synthesis_tRNA	tRNA-Met	SigA			
albG	+	3,64	Lifestyle_Biosynthesis of antibacterial compounds	Production of subtilosin; activity unknown	AbrB (-)	Rok (-)	ResD (+)	
ntdR	+	3,73	Lifestyle_Biosynthesis of antibacterial compounds	Activator of ntdABC-glcP operon; transcriptional regulator (LacI family)	AbrB (-)	NtdR (+)		
comK	+	2,74	Lifestyle_Genetic competence	Competence transcription factor	AbrB (-)	Spo0A (+)	CodY (-)	Rok (-), ComK (+), DegU (+)
comC	+	2,74	Lifestyle_Genetic competence	required for processing and translocation of ComGC, ComGD, ComGE and ComGG; protease	ComK (+)			
kre	+	3,31	Lifestyle_Genetic competence	inhibitor of competence; ComK repressor	ComK (-)			
sda	+	2,68	Lifestyle_Sporulation	checkpoint coupling replication and sporulation; inhibitor of Spo0A phosphorelay	LexA (-)	DnaA (+)		
yhfM	+	2,70	Lifestyle_Sporulation	U	SigF			
phrE	+	3,48	Lifestyle_Sporulation	Regulator of RapE phosphatase	CcpA (-)	AbrB (-)	SigH	CodY (-), ComA (+)
yozQ	+	3,61	Lifestyle_Sporulation	U	SigG			
cotM	+	3,87	Lifestyle_Sporulation	spore resistance; spore coat protein	SigE	GerE (+)	SpoIIID (-)	
yknT	+	3,88	Lifestyle_Sporulation	spore protection; spore coat protein	SigE	SpoIIID (+)		
ypzA	+	4,13	Lifestyle_Sporulation	U	SigG			
yisI	+	4,16	Lifestyle_Sporulation	inhibition of phosphorelay; Spo0A-P phosphatase	PchR (+)			
cotX	+	5,96	Lifestyle_Sporulation	spore resistance; spore crust assembly	SigE	GerE (+)	SpoIIID (-)	
cotC	+	6,28	Lifestyle_Sporulation	spore resistance; spore coat protein	SigK	GerE (+)	SpoIIID (-)	
yckD	+	6,38	Lifestyle_Sporulation	U	SigF/G			
yhcN	+	3,05	Lifestyle_Sporulation_germination	efficient spore germination; activity unknwon	SigF/G			
gerD	+	3,37	Lifestyle_Sporulation_germination	Scaffold of the germinosome	SigF/G			
sinR	+	2,76	Lifestyle_stationnary phase adaptation	Transcriptional regulator of post-exponential-phase responses genes, control of biofilm formation	AbrB (-)	SinR (-)	Spo0A (+) ScoC (-)	
abrB	+	4,34	Lifestyle_stationnary phase adaptation	Tansition state regulator_trnascritonal regulator	AbrB (-)	Spo0A (-)		
lrpB	+	3,51	Metabolism_Biosynthesis & acquisition of Aminoacids	metabolism of glycine; Repression of glyA transcription and KinB-dependent sporulation	U			
lrpA	+	5,26	Metabolism_Biosynthesis & acquisition of Aminoacids	metabolism of glycine; Repression of glyA transcription and KinB-dependent sporulation	U			
pyrR	+	3,56	Metabolism_Biosynthesis & acquisition of nucleotides	Transcriptional antiterminator of the pyr operon	PyrR			
pyrP	+	4,13	Metabolism_Biosynthesis & acquisition of nucleotides	uracil permease	PyrR			
tnrA	+	2,45	Metabolism_nitrogen metabolism	control of nitrogen assimilation; transcriptional regulator (MerR family)	GlnR (-)	TnrA (+)		
nupG	+	2,78	metabolism_nucelotide metabolism	Purine nucleosides transporter	G-box			
degR	+	3,58	Metabolism_Regulation of degradative enzyme production	control of DegU; Positive effector of DegU-phosphate stability	SigD			
rapI	+	2,96	Prophages & mobile genetic elements_ICEBs1	Control of ICEBs1, ImmR antagonist; response regulator aspartate phosphatase	ComA (+)	SigH		
ligB	+	2,96	Prophages & mobile genetic elements_ICEBs1	phage SP-beta replication_DNA ligase	U			
int	+	3,32	Prophages & mobile genetic elements_ICEBs1	Excision of the conjugative transposon ICEBs1; integrase	U			
immA	+	3,40	Prophages & mobile genetic elements_ICEBs1	control of ImmR activity; site-specific protease	U			
ydcO	+	3,42	Prophages & mobile genetic elements_ICEBs1	U	ImmR (-)			
xis	+	3,54	Prophages & mobile genetic elements_ICEBs1	Excision of the conjugative transposon ICEBs1; excisionase	ImmR (-)			
ydcP	+	3,83	Prophages & mobile genetic elements_ICEBs1	Conjugation and replication of ICEBs1; helicase processivity factor	ImmR (-)			
immR	+	3,86	Prophages & mobile genetic elements_ICEBs1	Control of transfer of the mobile genetic element ICEBs1; Transcriptional repressor	U			
xre	+	3,83	Prophages & mobile genetic elements_PBSX prophage	regulation of PBSX prophage gene expression; Transcriptional repressor	U			
ybzH	+	3,84	Prophages & mobile genetic elements_Prophage 1	unknown; putative transcriptional regulator	U			
yqaS	+	2,78	Prophages & mobile genetic elements_Skin element	unknown; similar to terminase	SigD			
sknR	+	3,04	Prophages & mobile genetic elements_Skin element	repression of yqaF operon of the skin element; transcriptional repressor (Xre family)	U			
yqbC	+	3,68	Prophages & mobile genetic elements_Skin element	U	U			
yqbG	+	4,94	Prophages & mobile genetic elements_Skin element	U	U			
yoyH	+	2,34	Prophages & mobile genetic elements_SP-beta prophage	U	U			
yoqL	+	2,49	Prophages & mobile genetic elements_SP-beta prophage	U	U			
yomO	+	2,49	Prophages & mobile genetic elements_SP-beta prophage	U	U			
yorS	+	2,54	Prophages & mobile genetic elements_SP-beta prophage	U	U			
yotM	+	2,56	Prophages & mobile genetic elements_SP-beta prophage	U	CsoR (-)	SigE/K		
yorB	+	2,58	Prophages & mobile genetic elements_SP-beta prophage	U	LexA (-)			
yorE	+	2,61	Prophages & mobile genetic elements_SP-beta prophage	U	U			
yorM	+	2,64	Prophages & mobile genetic elements_SP-beta prophage	U	U			
yosT	+	2,70	Prophages & mobile genetic elements_SP-beta prophage	U	U			
yonT	+	2,72	Prophages & mobile genetic elements_SP-beta prophage	toxin	as-yonT			
yopC	+	2,72	Prophages & mobile genetic elements_SP-beta prophage	U	U			
yorJ	+	2,78	Prophages & mobile genetic elements_SP-beta prophage	U	U			
yonV	+	2,82	Prophages & mobile genetic elements_SP-beta prophage	U	U			
yopK	+	2,99	Prophages & mobile genetic elements_SP-beta prophage	U	U			
yopQ	+	3,04	Prophages & mobile genetic elements_SP-beta prophage	U	U			
yopA	+	3,08	Prophages & mobile genetic elements_SP-beta prophage	U	U			
yopE	+	3,10	Prophages & mobile genetic elements_SP-beta prophage	U	U			
yonI	+	3,14	Prophages & mobile genetic elements_SP-beta prophage	U	U			
yopP	+	3,17	Prophages & mobile genetic elements_SP-beta prophage	U	U			
yoyJ	+	3,26	Prophages & mobile genetic elements_SP-beta prophage	U	U			
yopR	+	3,26	Prophages & mobile genetic elements_SP-beta prophage	U	U			
yokB	+	3,47	Prophages & mobile genetic elements_SP-beta prophage	U	U			
yopM	+	3,52	Prophages & mobile genetic elements_SP-beta prophage	U	U			
yorC	+	3,54	Prophages & mobile genetic elements_SP-beta prophage	U	LexA (-)			

yokC	+	3,72	Prophages & mobile genetic elements_SP-beta prophage	U	U		
yoyI	+	3,72	Prophages & mobile genetic elements_SP-beta prophage	U	U		
yopN	+	3,73	Prophages & mobile genetic elements_SP-beta prophage	U	U		
yotE	+	3,75	Prophages & mobile genetic elements_SP-beta prophage	U	CsoR (-)	SigE/K	
yopO	+	4,12	Prophages & mobile genetic elements_SP-beta prophage	similar to transcriptional regulator (Xre family)	U		
yonS	+	4,23	Prophages & mobile genetic elements_SP-beta prophage	U	U		
yotD	+	4,25	Prophages & mobile genetic elements_SP-beta prophage	Unknown; similar to acyl-coA synthetase	CsoR (-)	SigE/K	
yopF	+	4,33	Prophages & mobile genetic elements_SP-beta prophage	U	U		
yonR	+	5,29	Prophages & mobile genetic elements_SP-beta prophage	Similar to transcriptional regulator (Xre family)	U		
bhlB	+	6,45	Prophages & mobile genetic elements_SP-beta prophage	U	U		
yosU	+	8,08	Prophages & mobile genetic elements_SP-beta prophage	U	U		
dps	+	3,21	stress response	Fe storage and stress resistance; miniferritin	SigB		
SR5	+	3,99	stress response_Anti-toxin	contro of BsrE toxin expression; Antisense RNA to bsrE	U		
ydjO	+	3,09	stress response_cell envelope stress resistance	U	SigW	SigE	
mgsR	+	2,86	Stress response_oxydative stress resistance	Transcriptional regulator of a subset of the SigB general stress regulon; protection vs oxidative stress	SigB	MgsR (+)	
yydJ	+	3,00	Stress response_resistance to antimicrobials	control of LiaR-LiaS activity; putative ABC transporter (ATP-binding)	AbrB (-)	Rok (-)	
sigX	+	3,07	Stress response_Resistance to antimicrobials	Resistance to cationic antimcrobial peptides; RNA polymerase ECF-type sigma factor	SigX	YvrbH (+)	
yydI	+	3,86	Stress response_resistance to antimicrobials	control of LiaR-LiaS activity; putative ABC transporter (permease)	AbrB (-)	Rok (-)	
yydG	+	4,50	Stress response_resistance to antimicrobials	control of LiaR-LiaS activity; oxidoreductase	AbrB (-)	Rok (-)	
yydH	+	4,86	Stress response_resistance to antimicrobials	control of LiaR-LiaS activity; putative ABC transporter	AbrB (-)	Rok (-)	
yobK	+	2,48	Stress response_toxins, antitoxins and immunity against toxins	antitoxin, inhibition of the cytotoxic activity of YobL	U		
yobL	+	2,78	Stress response_toxins, antitoxins and immunity against toxins	Toxin	U		
yxxD	+	2,92	Stress response_toxins, antitoxins and immunity against toxins	Antitoxin; inhibition of cytotxic YxiD	U		
sdpI	+	3,02	Stress response_toxins, antitoxins and immunity against toxins	protection against SdpC toxin	AbrB (-)	SdpR (-)	
sdpA	+	5,21	Stress response_toxins, antitoxins and immunity against toxins	maturation of SdpC toxin	AbrB (-)	Rok (-)	Spo0A (+)
ywnA	+	2,29	U	U	U		
yvkN	+	2,33	U	U	U		
yrkD	+	2,53	U	U	U		
yubF	+	2,54	U	U	U		
yceK	+	2,56	U	unknown; putative transcriptional regulator	U		
yozV	+	2,58	U	U	U		
yurQ	+	2,60	U	U	U		
ypbS	+	2,60	U	U	U		
ynaB	+	2,63	U	U	U		
ykvN	+	2,64	U	Unknown; MarR family transcription regulator	U		
yfnC	+	2,65	U	unknown; similar to fosmidmycin resistance protein	U		
ycxB	+	2,67	U	U	U		
yqzF	+	2,68	U	U	U		
ydeI	+	2,70	U	U	U		
ywpF	+	2,70	U	U	U		
ybfF	+	2,70	U	U	U		
yqgW	+	2,71	U	U	U		
yrdC	+	2,78	U	U	U		
yydD	+	2,80	U	U	U		
yoeC	+	2,81	U	U	U		
yfzA	+	2,96	U	U	U		
yezA	+	2,96	U	U	U		
yjfB	+	2,97	U	U	SigD		
ypoP	+	3,00	U	Similar to transcriptional regulator (MarR family)	U		
yqkE	+	3,03	U	U	U		
yraH	+	3,05	U	U	U		
pbuO	+	3,06	U	unknown; similar to permease PbuG	PurR (-)		
yncB	+	3,16	U	U	U		
ydeB	+	3,26	U	unknown; putative transcriptional regulator	U		
yeeD	+	3,27	U	U	U		
yaaC	+	3,28	U	putative sporulation protein	U		
ydfL	+	3,30	U	unknown; similar to multidrux efflux transporter regulator of MreR family	U		
ybfE	+	3,33	U	U	U		
ylbN	+	3,39	U	may be required for accumulation of 23S rRNA	U		
yybN	+	3,40	U	U	Rok (-)	DnaA (-)	
yxkC	+	3,43	U	U	SigD	TnrA (+)	
ynaE	+	3,49	U	U	U		
yxaC	+	3,50	U	Holin-like auxiliary protein	U		
yrzM	+	3,53	U	U	U		
ykzT	+	3,57	U	U	U		
ykvS	+	3,57	U	U	U		
yjiA	+	3,57	U	U	U		
ypzI	+	3,61	U	U	U		
yjzD	+	3,62	U	U	U		

yybM	+	3,66	U	U	Rok (-)	DnaA (-)
ymzD	+	3,68	U	U	U	
yoyA	+	3,68	U	U	U	
yobD	+	3,69	U	similar to transcriptional regulator (Xre family)	U	
yneF	+	3,73	U	U	Spo0A (+)	
yddK	+	3,76	U	U	U	
yugI	+	3,81	U	U	RelA	
yybL	+	3,82	U	similar to ABC transporter (permease)	Rok (-)	DnaA (-)
yozF	+	3,87	U	U	U	
ybeF	+	3,90	U	putative sporulation protein	U	
ygzA	+	4,10	U	U	U	
yoaQ	+	4,16	U	U	U	
ydzP	+	4,22	U	U	U	
yxlA	+	4,32	U	similar to purine-cytosine permease	U	
yjzI	+	4,47	U	U	U	
yabE	+	4,50	U	unknown; Similar to CW binding prot	sRNA(-)	
ykzB	+	4,99	U	U	TnrA (+)	PhoP (+)
yocC	+	5,47	U	U	U	
yozH	+	5,77	U	U	U	
ykoL	+	6,46	U	U	PhoP (+)	TnrA (+)
ydcG	+	17,56	U	U	U	
ydcF	+	71,73	U	U	U	
yvzA	+	2,72	U (put based on regulation: lifestyle_transition state)	U	AbrB (-)	DegU (+)
yfmI	+	2,74	U (put based on regulation: lifestyle_transition state)	unknown, similar to macrolide-efflux transporter	AbrB (-)	Spo0A (+)
ydeH	+	2,89	U (put based on regulation: lifestyle_transition state)	U	AbrB (-)	
yvnA	+	2,97	U (put based on regulation: lifestyle_transition state)	U	AbrB (-)	CcpA (-)
yjcM	+	3,91	U (put based on regulation: lifestyle_transition state)	U	AbrB (-)	SigD
yjdG	+	4,87	U (put based on regulation: lifestyle_transition state)	U	AbrB (-)	
yozL	+	14,32	U	U	LexA (-)	
